# Establishment of an age‐ and tumor microenvironment‐related gene signature for survival prediction in prostate cancer

**DOI:** 10.1002/cam4.4776

**Published:** 2022-05-09

**Authors:** Lei Chen, Meng Zhang, Jun Zhou, Li Zhang, Chaozhao Liang

**Affiliations:** ^1^ Department of Urology The First Affiliated Hospital of Anhui Medical University Hefei China; ^2^ Institute of Urology Anhui Medical University Hefei China; ^3^ Anhui Province Key Laboratory of Genitourinary Diseases Anhui Medical University Hefei China; ^4^ Anhui Institute of Translational Medicine Hefei China

**Keywords:** age, gene signature, nomogram, prostate cancer, tumor microenvironment

## Abstract

**Background:**

The incidence of prostate cancer (PCa) increases with age, and age and tumor microenvironment (TME) have important roles in the development of PCa, while the underlying mechanisms have not been fully elucidated.

**Materials and method:**

The Cancer Genome Atlas‐Prostate Adenocarcinoma (TCGA‐PRAD) RNA‐Seq, the Surveillance, Epidemiology, and End Results (SEER‐PRAD), and ESTIMATE data were downloaded, and the clinical information of PRAD patients in our cohort was collected. The associations among age, TME, and PCa were analyzed. The age‐ and TME‐related risk score (ATRS) of each TCGA‐PRAD sample was calculated based on the identified age‐ and TME‐related differentially expressed genes (DEGs), and the correlation of ATRS with immune‐related characteristics of PCa patients was analyzed, and the ATRS‐based overall survival (OS)‐predicting nomogram was also established.

**Results:**

Age was correlated with OS, PSA level, tumor stage, T stage, N stage, Gleason score, nerve invasion of PCa, and age was positively correlated with stromal, immune, and ESTIMATE scores. The compositions of immune cells of TCGA‐PRAD patients altered with age. Nine age‐ and TME‐related prognostic DEGs were identified, and the ATRS of each TCGA‐PRAD patient was calculated based on the identified nine DEGs. The ATRS was associated with the expression of immune checkpoints and intratumoral cytolytic activity, and the ATRS‐based nomogram performed well in predicting the outcomes of PCa patients.

**Conclusions:**

Age and TME had crucial roles in PCa, and the ATRS gene signature was associated with the immune‐related characteristics of PCa patients, which showed good performance in predicting OS of PCa patients.

## Graphical abstract

In this study, we found that age was correlated with the clinical characteristics and tumor microenvironment of PCa patients, and the age and tumor microenvironment ‐ related gene signature (ATRS) and the ATRS‐based nomogram were established to predict the prognosis of PCa patients.

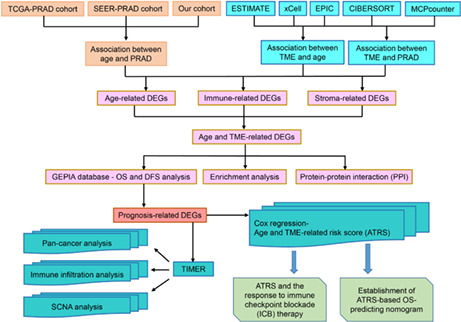

## 1 INTRODUCTION

As one of the most common malignancies, approximately 192,000 new cases and 33,000 deaths of prostate cancer (PCa) were estimated to occur in America in 2020.^1^ With the arrival of the population aging and the wide application of PSA‐based screening, the incidence of PCa increased and the risk of PCa‐related mortality and metastasis declined,^2^ and the median age was 66 years at diagnosis.^3^ In 2017, PCa was the third and second‐leading cancer‐related death inpatients aged 60–79 years and ≥80 years in America, respectively.^1^ The treatment for PCa has advanced dramatically, and radical prostatectomy (RP) and radiotherapy (RT) were regarded as the first‐line treatment options for patients at the early stage, while for metastatic PCa (mPCa) patients, chemotherapy, immunotherapy, hormonal and radionuclide therapy were recommended.^4^, ^5^ For PCa patients received androgen‐deprivation therapy (ADT), age was an independent factor of overall survival (OS) and disease‐specific survival (DSS).^6^ Hence, age may exert crucial role in guiding the diagnosis and treatment of PCa, and the underlying mechanisms deserved further investigation.

The tumor microenvironment (TME) is comprised of various components, including stromal cells, immune cells, and extracellular matrix, which participate in the metastasis, immune escape, and treatment response of the tumor.^7^, ^8^ Treatment strategies targeting TME emerge as novel approaches to cancer therapy, including inhibiting neovascularization, enhancing the anti‐tumor activity of the immune system, etc.^9^ Moreover, the responses to immune checkpoint blockade (ICB) therapy were modulated by TME, and the TME markers were associated with the response and resistance to ICB therapy.^10^ In PCa, patients with different levels of immune cell infiltration have different survival times,^11^ and the immune scores quantified by the ESTIMATE algorithm were associated with immune infiltration and the OS of PCa patients.^12^ Immunosenescence is characterized by the aging of the immune system accompanied by a decline in its function in the elderly, which caused abnormalities of the immune response and elicited the development of malignancies.^13^ With age, the immune cell subsets and the function of immune cells alter, leading to the attenuation in immune surveillance.^13^, ^14^ Furthermore, the rearrangement of ECM in the aged TME disrupts its integrity and accelerates the initiation and metastasis of the tumor.^15^ Hence, the aged TME may have significant effects on the development of PCa.

In this study, we explored the associations among age, TME, and the clinical characteristics of PCa patients in three cohorts. The landscape of immune cells in the TCGA‐PRAD samples was estimated by four databases, and the alterations of immune cell compositions with age were analyzed. The age‐ and TME‐related differentially expressed genes (DEGs) were identified, and the age‐ and TME‐related risk score (ATRS) and ATRS‐based nomogram were established, which were associated with the outcomes of PCa patients.

## 2 MATERIALS AND METHODS

### 2.1 Datasets

**FIGURE 1:**
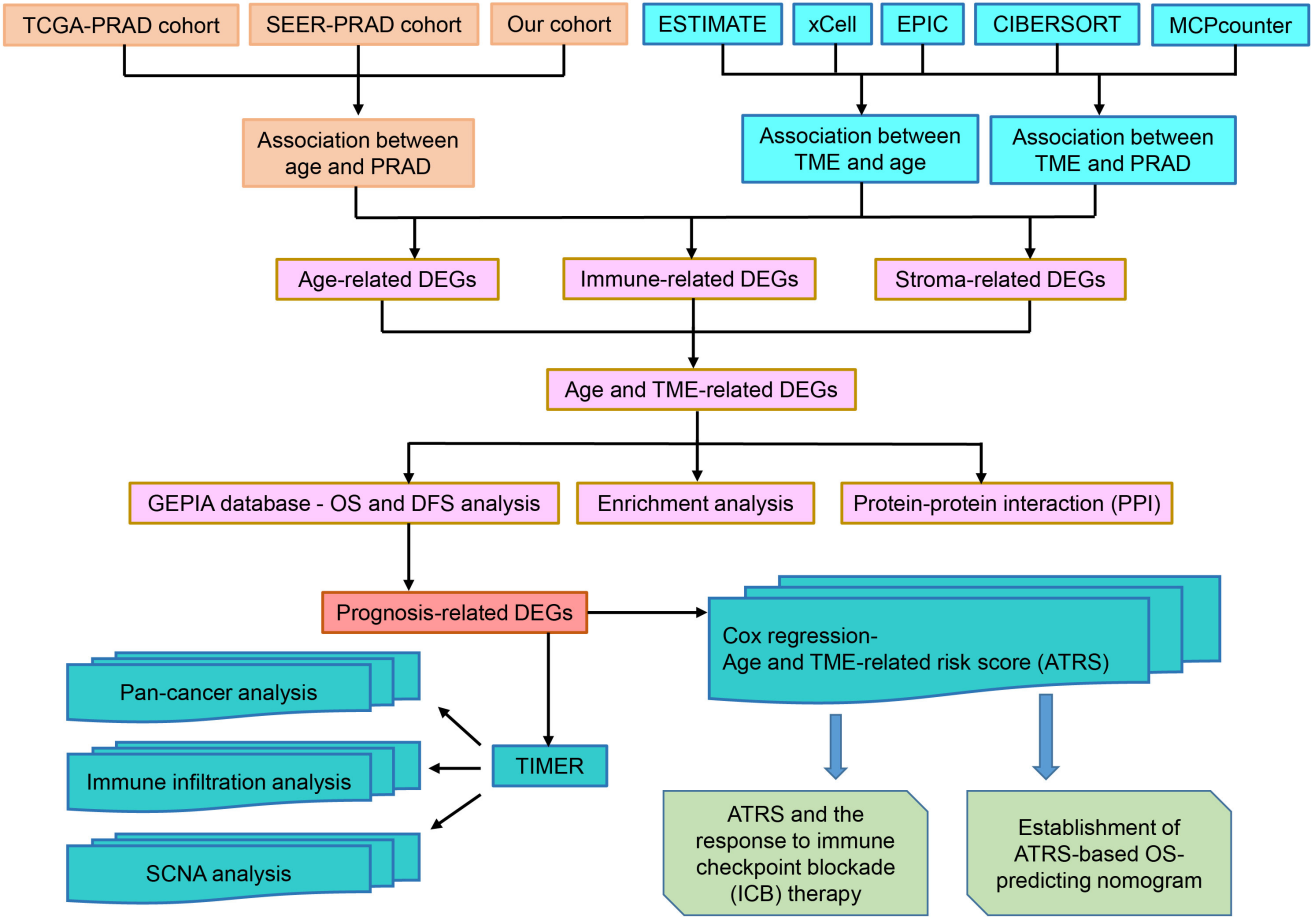
The flowchart for establishing the age‐ and TME‐related gene signature of prostate cancer in the study. DEGs, differentially expressed genes; DFS, Disease‐Free Survival; OS, overall survival; PRAD, Prostate Adenocarcinoma; SCNA, somatic copy number alteration; SEER, Surveillance, Epidemiology, and End Results; TCGA, The Cancer Genome Atlas; TME, tumor microenvironment

All the procedures are displayed in the flowchart (Figure 1). The Cancer Genome Atlas‐Prostate Adenocarcinoma (TCGA‐PRAD) RNA‐Seq data and the corresponding clinical data were downloaded from the TCGA database on May 20, 2021. The Surveillance, Epidemiology, and End Results (SEER)‐9 Registry Research Data of PRAD were obtained from the SEER Program by the SEER*Stat software (version 8.3.9). A cohort of pathologically diagnosed PRAD patients aged >18 years from the First Affiliated Hospital of Anhui Medical University was enrolled in the current study between January 2015 and August 2021, which was approved by the committee of The First Affiliated Hospital of Anhui Medical University, and an exempt of written informed consent from patients was granted by the Ethics Committee for this purpose (PJ 2021‐14‐23). In our cohort, PCa patients' information including age, body mass index (BMI), total prostate‐specific antigen (PSA) level, Gleason score, primary Gleason score, invasion of nerve, and seminal vesicle, T stage were collected.

### 2.2 Association among age, TME, and PCa

The PCa patients in TCGA, SEER, and our cohorts were divided into two groups: age < 60 years and age ≥ 60 years, and the clinical characteristic data in these two groups were compared by *t*‐test and *χ* ^2^ test. In the TCGA‐PRAD cohort, the positive lymph node ratio (PLNR) was calculated based on the number of positive lymph nodes and the total examined lymph nodes. The ESTIMATE algorithm was used to calculate stromal, immune, and estimate score of each TCGA‐PRAD sample.^16^ The proportion or abundance of immune cells in each TCGA‐PRAD sample was quantified by using the CIBERSORT,^17^ MCPcounter,^18^ EPIC,^19^ and xCell^20^ methods. The distributions of immune cells in different age groups were compared by Mann–Whitney *U* test.

### 2.3 Identification of age‐ and TME‐related differentially expressed genes

The DEGs (*P*adj < 0.05, logFC > 1 or logFC < −1) in different age groups (<60 years and ≥60 years), immune and stromal score groups (<median score and ≥median score) were identified by using ‘*edge’* package in R software (version 3.6.3), and common DEGs were obtained through the intersection of age‐ and TME‐related DEGs, and visualized by Venn diagram.

### 2.4 Selection of prognostic DEGsand establishment of age‐ and TME‐related gene signature

The protein–protein interaction (PPI) network was established to visualize the correlation among common DEGs by STRING database^21^ and Cytoscape software (version 3.7.1). The biological function of these common DEGs was annotated by the Metascape tool (https://metascape.org/).^22^ The GEPIA tool^23^ was used to explore the effects of these common DEGs on the OS and Disease‐Free Survival (DFS) of PCa to identify prognosis‐related DEGs, and the pearson correlation coefficients among these genes were calculated. The pan‐cancer analysis was performed to analyze the expressions of the identified prognosis‐related DEGs by the Tumor IMmune Estimation Resource (TIMER) tool,^24^, ^25^ which was also applied to explore the correlation of the identified prognosis‐related DEGs with immune infiltration of PCa. The correlation of immune infiltration with the somatic copy number alterations (SCNAs) of the identified genes was also analyzed by TIMER. The age‐ and TME‐related risk score (ATRS) of each TCGA‐PRAD sample was calculated based on the expression levels of the prognosis‐related DEGs and the corresponding coefficients, which were expressed as follows: $\text{ATRS}=\sum_{i=1}^{n}\;\left({\text{coef}}_{i}\times {\text{Expr}}_{i}\right)$, and PCa patients were divided into high and low ATRS groups based on the median value of the ATRS gene signature.

### 2.5 Estimation of ATRSand the response to immunotherapy

PCa patients' responses to ICB therapy were assessed by the Immune Cell Abundance Identifier (ImmuoCellAl) method,^26^ and the correlations of ATRS with the expression levels of 34 types of immune checkpoints were analyzed. The immunophenoscore (IPS) of each TCGA‐PRAD patient was obtained from The Cancer Immunome Atlas (TCIA) database^27^ to estimate patients' responses to anti‐PD‐1 and anti‐CTLA‐4 therapies. The CYT scores of TCGA‐PRAD samples were calculated to quantify the immune cytolytic activities of the intratumoral immune infiltration, additionally, tumor mutations, predicted, and observed neoantigens were also quantified.^28^ The correlations of ATRS with IPS, tumor mutation burdens, neoantigens, and the response to ICBs were assessed to explore the predictive value of ATRS on immunotherapy response.

### 2.6 Construction of the ATRS‐based OS‐predicting nomogram

The predictive nomogram based on the ATRS gene signature was established and visualized by using the “survival” and “regplot” packages in R software, and the decision curve analysis and calibration curve were drawn by using the “ggDCA” and “rms” packages in R.

### 2.7 Statistics analysis

Statistics analysis was performed by using R software (version 3.6.3) and SPSS software (version 22.0). Data are presented as the mean ± standard deviation (mean ± SD) and count (%) for normally distributed continuous variables and categorical variables, respectively. The Kolmogorov–Smirnov test was used to assess the normality of the data. The *t*‐test, Mann–Whitney *U* test, Kruskal–Wallis, one‐way ANOVA, Fisher's exact tests, and χ^2^ tests were used to examine the differences between groups for continuous and categorical variables, respectively, and *p* (two‐sides) <0.05 was considered statistically significant.

## 3 RESULTS

### 3.1 Age was associated with PCa patients' clinical characteristics

**FIGURE 2:**
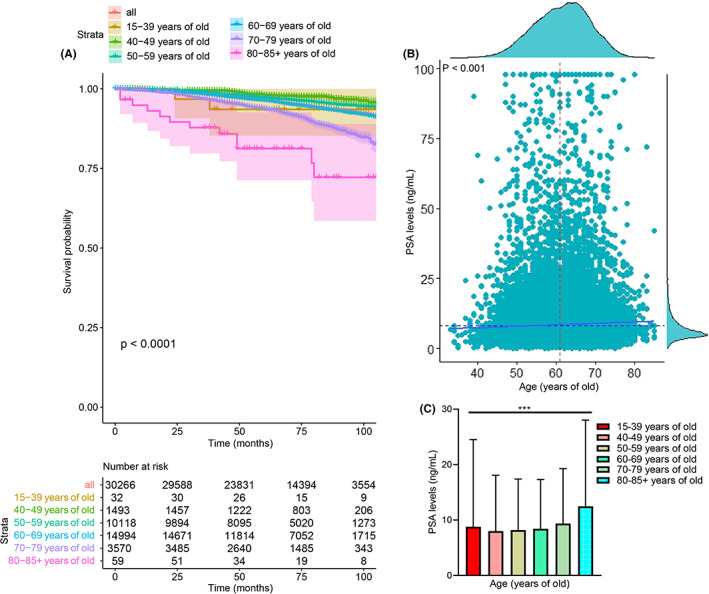
Age was associated with overall survival (OS) and PSA levels of PCa patients in SEER‐PRAD cohort. The survival time of PCa patients declined with age (A), and the PSA levels increased with age (B, C)

As shown in Figure 2A–C, PCa patients at higher age stages had poorer OS compared with younger patients in the SEER database, and PSA levels increased with age. The associations between age and PCa patients' clinical characteristic data in TCGA‐PRAD, SEER, and our cohorts were analyzed. As shown in Table 1, age was associated with T stage, N stage, PLNR, Gleason score, and primary Gleason score in TCGA‐PRAD cohort (all *p* < 0.05). In the SEER database, age was correlated with race, tumor stage, T stage, N stage, Gleason score, PSA level (Table S1, all *p* < 0.01), and in our cohort, age was associated with nerve invasion (Table S2, *p* < 0.01). Therefore, PCa patients at different age stages have different clinical characteristics.

**TABLE 1:** Characteristics of prostate cancer patients at different age stages in TCGA‐PRAD cohort

Characteristic	Age (years)		Total	χ^2^	*p*
	<60 (*n* = 201)	≥60 (*n* = 294)			
	*n* = 201	*n* = 294	495		
Race, no. (%)				15.118	**0.004**
Black/African American	35 (17.4)	21 (7.1)	56		
White	153 (76.1)	259 (88.1)	412		
Asian	6 (3.0)	6 (2.0)	12		
American Indian/Alaska Native	1 (0.5)	0 (0)	1		
Unknown	6 (3.0)	8 (2.7)	14		
Clinical T stage, no. (%)				6.626	**0.036**
T1 + T2	165 (82.1)	215 (73.1)	380		
T3 + T4	36 (17.9)	76 (25.9)	112		
Unknown	0 (0)	3 (1.0)	3		
Clinical N stage, no. (%)				7.833	**0.020**
N0	128 (63.7)	216 (73.5)	344		
N1	33 (16.4)	45 (15.3)	78		
Unknown	40 (19.9)	33 (11.2)	73		
Clinical M stage, no. (%)				1.214	0.545
M0	185 (92.0)	268 (91.2)	453		
M1	2 (1.0)	1 (0.3)	3		
Unknown	14 (7.0)	25 (8.5)	39		
Pathological T stage, no. (%)				9.364	**0.009**
T1 + T2	92 (45.8)	95 (32.3)	187		
T3 + T4	106 (52.7)	195 (66.3)	301		
Unknown	3 (1.5)	4 (1.4)	7		
Pathological N stage, no. (%)				7.833	**0.020**
N0	128 (63.7)	216 (73.5)	344		
N1	33 (16.4)	45 (15.3)	78		
Unknown	40 (19.9)	33 (11.2)	73		
PLNR, no. (%)				11.504	**0.003**
<10%	128 (63.7)	223 (75.9)	351		
≥10%	22 (10.9)	31 (10.5)	53		
Unknown	51 (25.4)	40 (13.6)	91		
Residual tumor, no. (%)				2.354	0.308
R0	131 (65.2)	183 (62.2)	314		
R1 + R2	55 (27.4)	96 (32.7)	151		
Rx + unknown	15 (7.5)	15 (5.1)	15		
Gleason score, no. (%)				7.610	**0.006**
≤7	133 (66.2)	158 (53.7)	291		
>7	68 (33.8)	136 (46.2)	204		
Primary Gleason score, no. (%)				18.504	**<0.001**
≤3	103 (51.2)	94 (32.0)	197		
>3	98 (48.8)	200 (68.0)	298		

Bold indicates statistically significant value (*p* < 0.05).

Abbreviation: PLNR, positive lymph node ratio.

### 3.2 TMEwas associated with PCapatients' age and clinical characteristics

**FIGURE 3:**
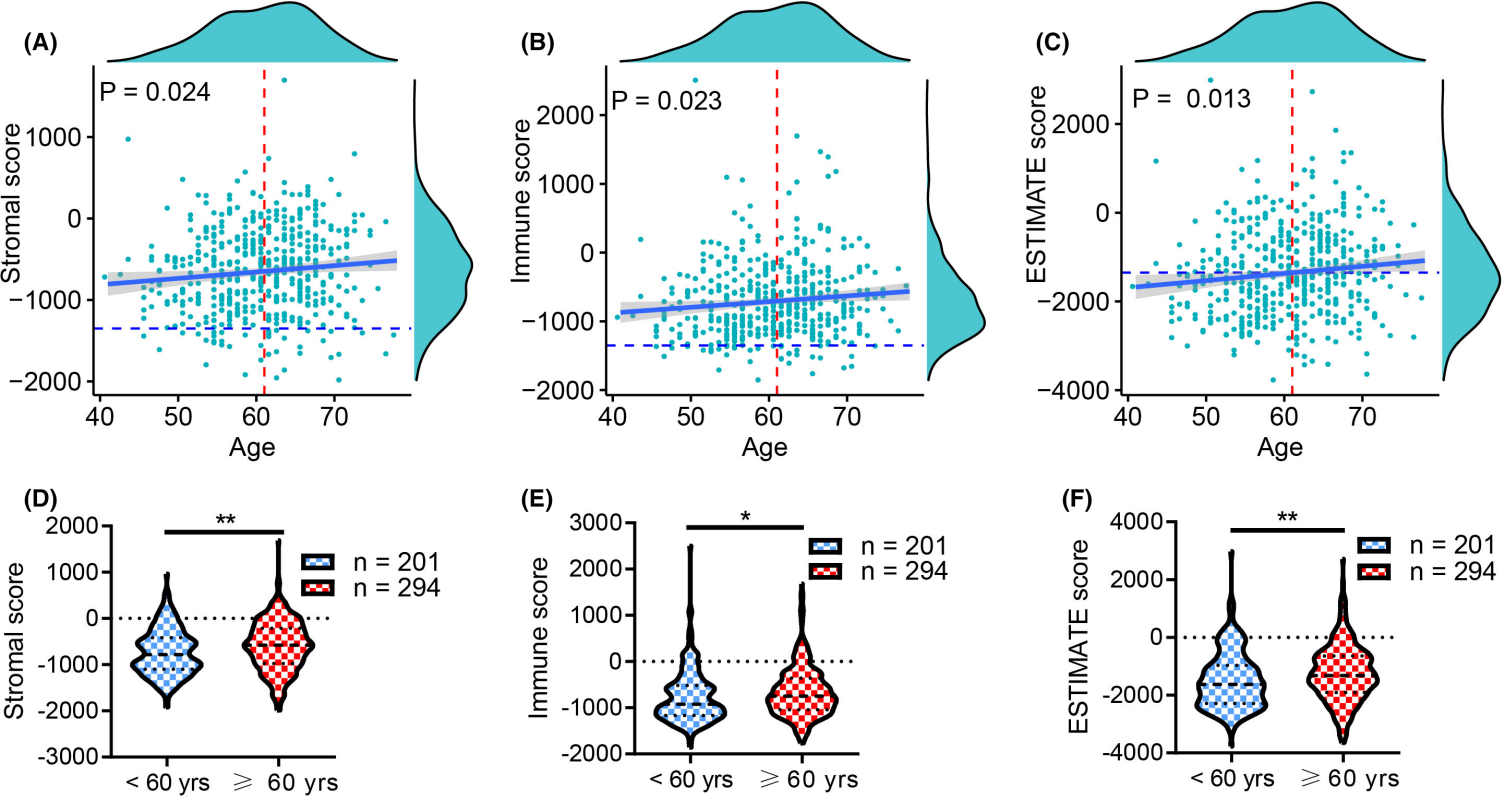
Age was associated with stromal, immune, and ESTIMATE scores of PCa patients. The stromal, immune, and ESTIMATE scores were increased with age (A–C), and PCa patients aged ≥60 years had higher stromal, immune, and ESTIMATE scores (D–F)

**FIGURE 4:**
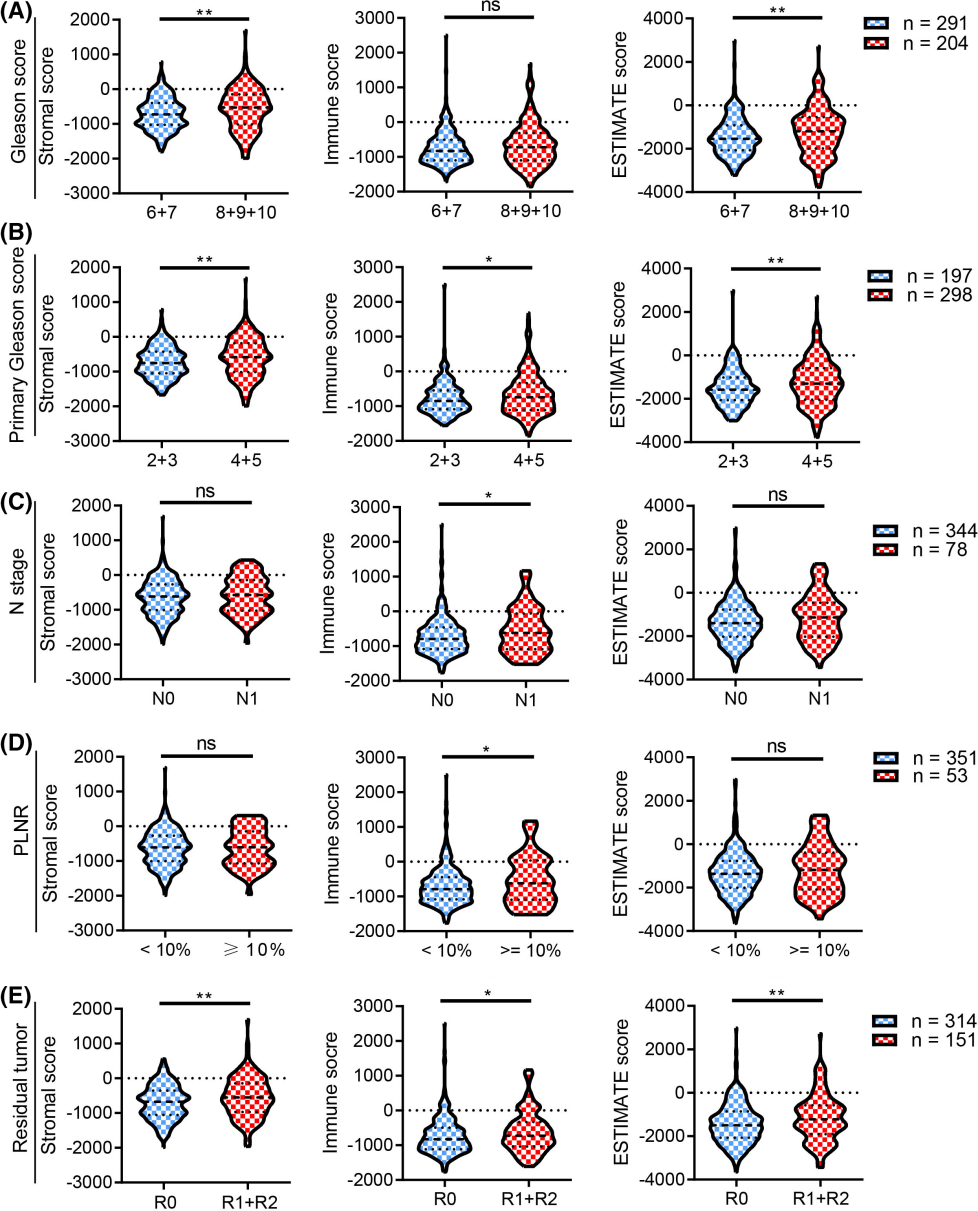
The stromal, immune, and ESTIMATE scores were associated with clinical characteristics of PCa patients. PCa patients with higher Gleason score (A), primary Gleason score (B), N stage (C), PLNR (D), and residual tumor (E) had higher stromal, immune, or ESTIMATE scores

The association between TME and PCa patients' age and clinical characteristics was determined. We found that age was positively correlated with the stromal score, immune score, and ESTIMATE score (all *p* < 0.05, Figure 3A–C), and PCa patients aged ≥60 years have higher stromal, immune, and ESTIMATE scores than patients <60 years (all *p* < 0.05, Figure 3D–F). PCa patients with higher Gleason scores had higher stromal and ESTIMATE scores (all *p* < 0.01, Figure 4A), and patients with higher primary Gleason scores had higher stromal, immune, and ESTIMATE scores (all *p* < 0.05, Figure 4B). Additionally, patients with lymph node (LN) metastasis had higher immune scores (*p* < 0.05, Figure 4C), and we found that PCa patients with the PLNR of ≥10% had higher immune scores (*p* < 0.05, Figure 4D). PCa patients with residual tumor (R1 + R2) had higher stromal, immune, and ESTIMATE scores (all *p* < 0.05, Figure 4E) than patients with R0.

### 3.3 Association between age and immune infiltration levels of PCa

**FIGURE 5:**
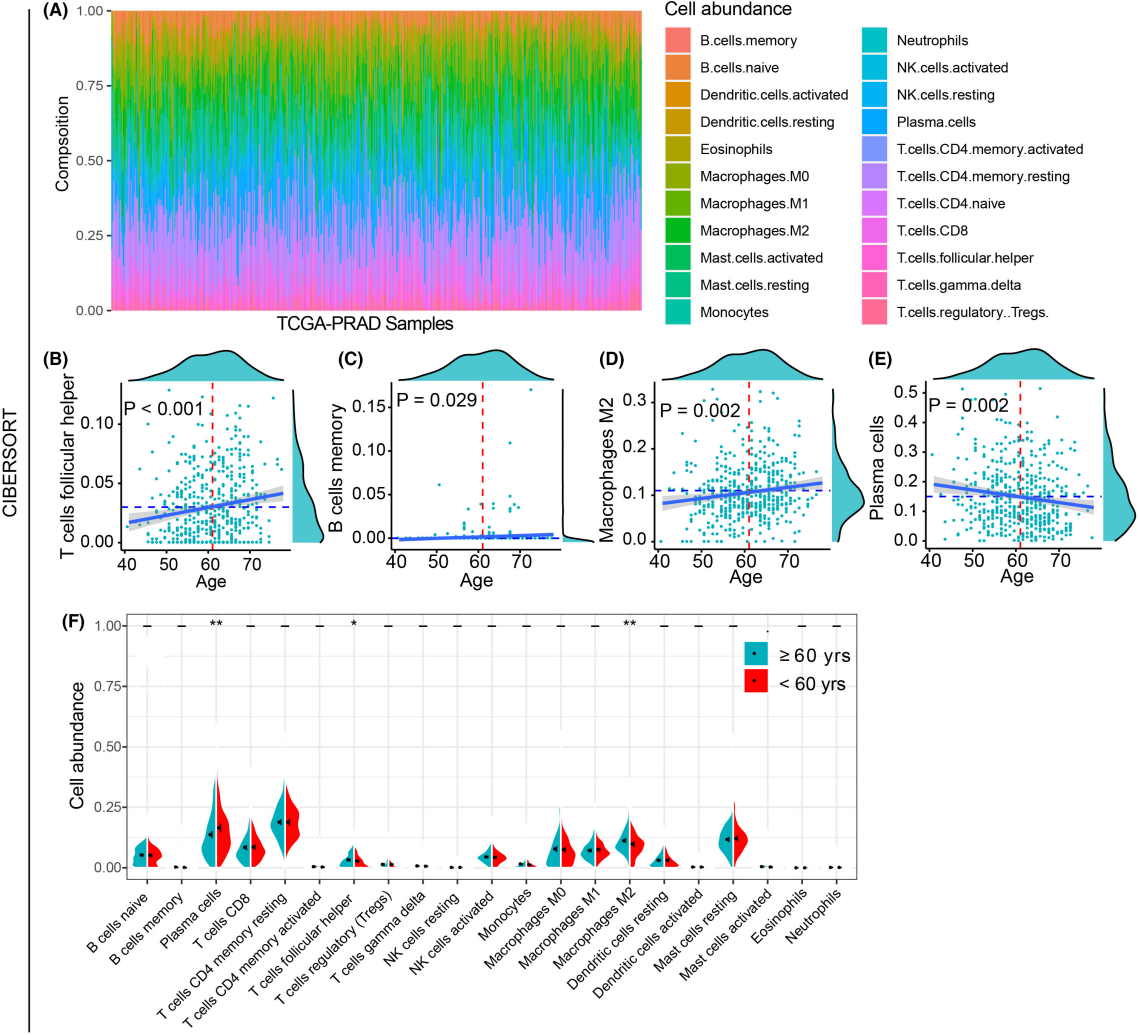
The alterations of immune cell infiltration in TCGA‐PRAD samples by using CIBERSORT. The histogram showed the composition of 22 types of immune cells in each TCGA‐PRAD sample (A). Age was positively correlated with the proportions of T cells follicular helper, B cells memory, and macrophages M2 (B–D), while negatively associated with plasma cells (E). The levels of T cells follicular helper and macrophage M2 cells were elevated in patients aged ≥60 years, whereas the levels of plasma cells were decreased in patients aged ≥60 years (F)

We further explored the immune cells alterations of TME in PCa patients at different age stages by using four databases, including CIBERSORT, EPIC, MCPcounter, and xCell. In CIBERSORT database, the composition of 22 types of immune cell in each TCGA‐PRAD sample was displayed in Figure 5A, and age was positively correlated with the levels of T cells follicular helper, B cells memory, and macrophages M2, while negatively associated with plasma cells (all *p* < 0.05, Figure 5B–E). The levels of T cells follicular helper and macrophage M2 cell was elevated in PCa patients aged ≥60 years, whereas the level of plasma cell was decreased in PCa patients aged ≥60 years (all *p* < 0.05, Figure 5F). In EPIC database, the composition of eight types of immune cell in each TCGA‐PRAD sample was displayed in Figure S1A, and age was negatively associated with CD4^+^T cells and CD8^+^T cells (all *p* < 0.01, Figure S1B,C), while positively correlated with macrophages (*p* = 0.001, Figure S1D). The levels of cancer‐associated fibroblasts (CAFs) and macrophages were elevated in PCa patients aged ≥60 years, whereas the levels of CD4^+^T cells and CD8^+^T cells were decreased in PCa patients aged ≥60 years (all *p* < 0.05, Figure S1E). In MCPcounter database, the composition of 10 types of immune cells in each TCGA‐PRAD sample was displayed in Figure S1F, and age was negatively associated with monocytic lineage and neutrophils (all *p* < 0.01, Figure S1G,H). The levels of monocytic lineage and neutrophils cells were decreased in PCa patients aged ≥60 years (all *p* < 0.05, Figure S1I). In xCell database, the composition of 64 types of immune cell in each TCGA‐PRAD sample was displayed in Figure S2A, and age was positively correlated with the levels of aDCs, adipocytes, astrocytes, mesangial cells, pro B cells, Th2 cells, and Tgd cells, while negatively associated with myocytes, platelets, and smooth muscle (all *p* < 0.05, Figure S2B–K). The levels of adipocytes, astrocytes, chondrocytes, macrophages M1, mesangial cells, monocytes, pericytes, and aDC were elevated in PCa patients aged ≥60 years, whereas the levels of myocytes and platelets were decreased in PCa patients aged ≥60 years (all *p* < 0.05, Figure S2L–N).

### 3.4 Identified age‐ and TME‐related DEGs

**FIGURE 6:**
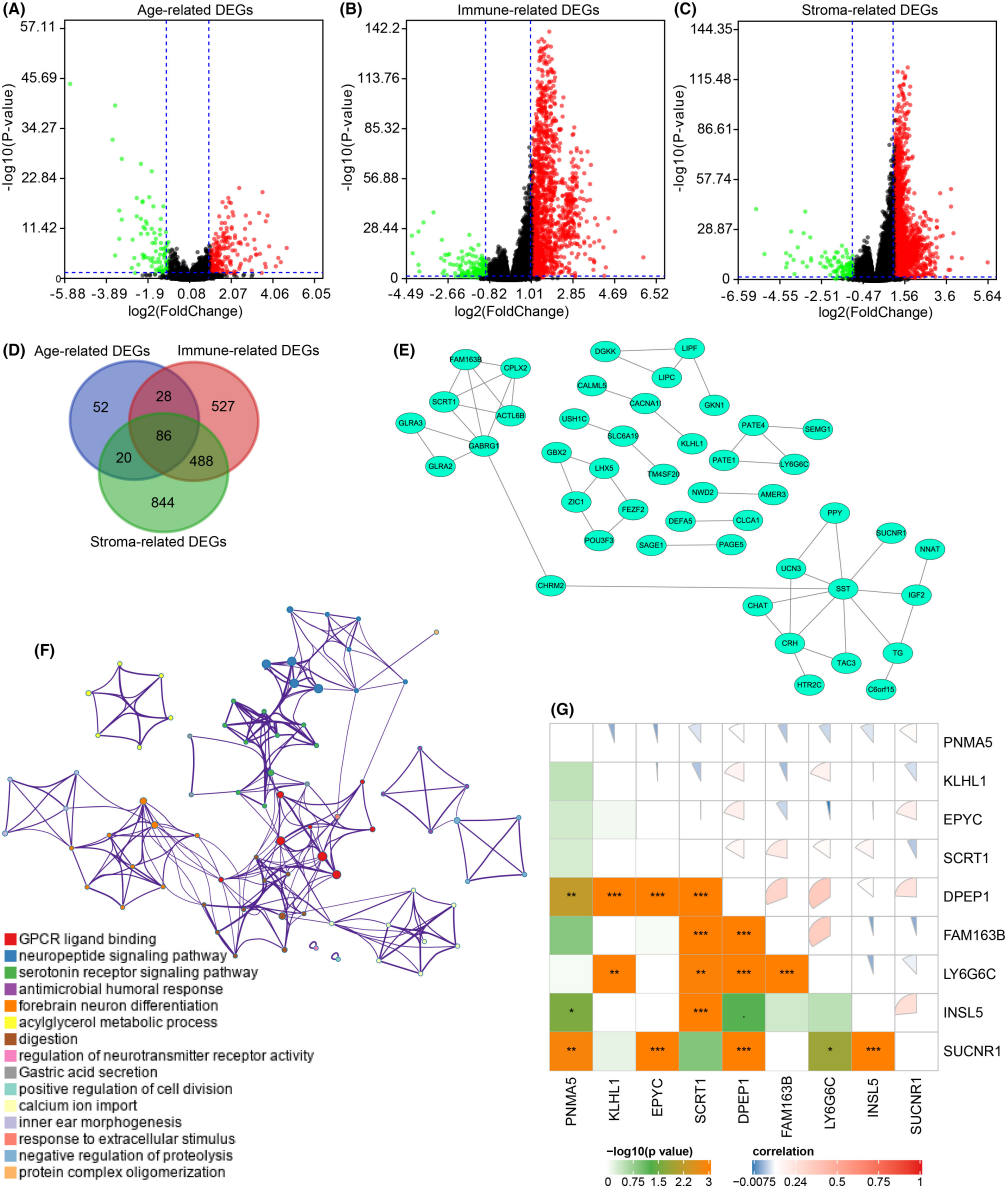
Identification of age‐ and TME‐related DEGs. The volcano plots showed age, immune, and stromal‐related DEGs, respectively (A–C), and 86 age‐ and TME‐related DEGs were selected (D). The protein–protein interaction network displayed the association between age‐ and TME‐related DEGs (E). The network indicated the enriched terms of the 86 age‐ and TME‐related DEGs (F). The pie plot indicated the correlation between the nine age‐ and TME‐related DEGs (G)

The three volcano plots displayed the age‐ and TME‐related DEGs (Figure 6A–C). The Venn diagram showed the common 86 DEGs (Figure 6D), and the protein–protein interaction (PPI) network indicated the interaction among these common DEGs (Figure 6E). Furthermore, these common DEGs were involved in several important biological processes, including GPCR ligand binding, positive regulation of cell division, etc. (Figure 6F). To identify prognosis‐related DEGs, we performed OS and DFS analysis for the 86 identified genes, and we found that nine genes (EPYC, FAM163B, INSL5, SUCNR1, SCRT1, DPEP1, KLHL1, LY6G6C, and PNMA5) were associated with OS and DFS of PCa patients (Figure S3A–I). Hence, these nine genes may have significant roles in the prognosis of PCa. We further performed pan‐cancer analysis and found that these nine genes were overexpressed in most cancer types (Figures S4 and S5). Additionally, most of these nine genes were correlated with the immune infiltration of PCa (Figures S6 and S7), and the SCNAs of these nine genes were also associated with immune filtration of PCa (Figure S8 and S9). Therefore, the nine identified genes may have significant effects on the survival and immune infiltration of PCa, and the correlation plot further displayed the associations among these nine prognosis‐related DEGs (Figure 6G).

### 3.5 Establishment of age‐ and TME‐related gene signature in PCa

**FIGURE 7:**
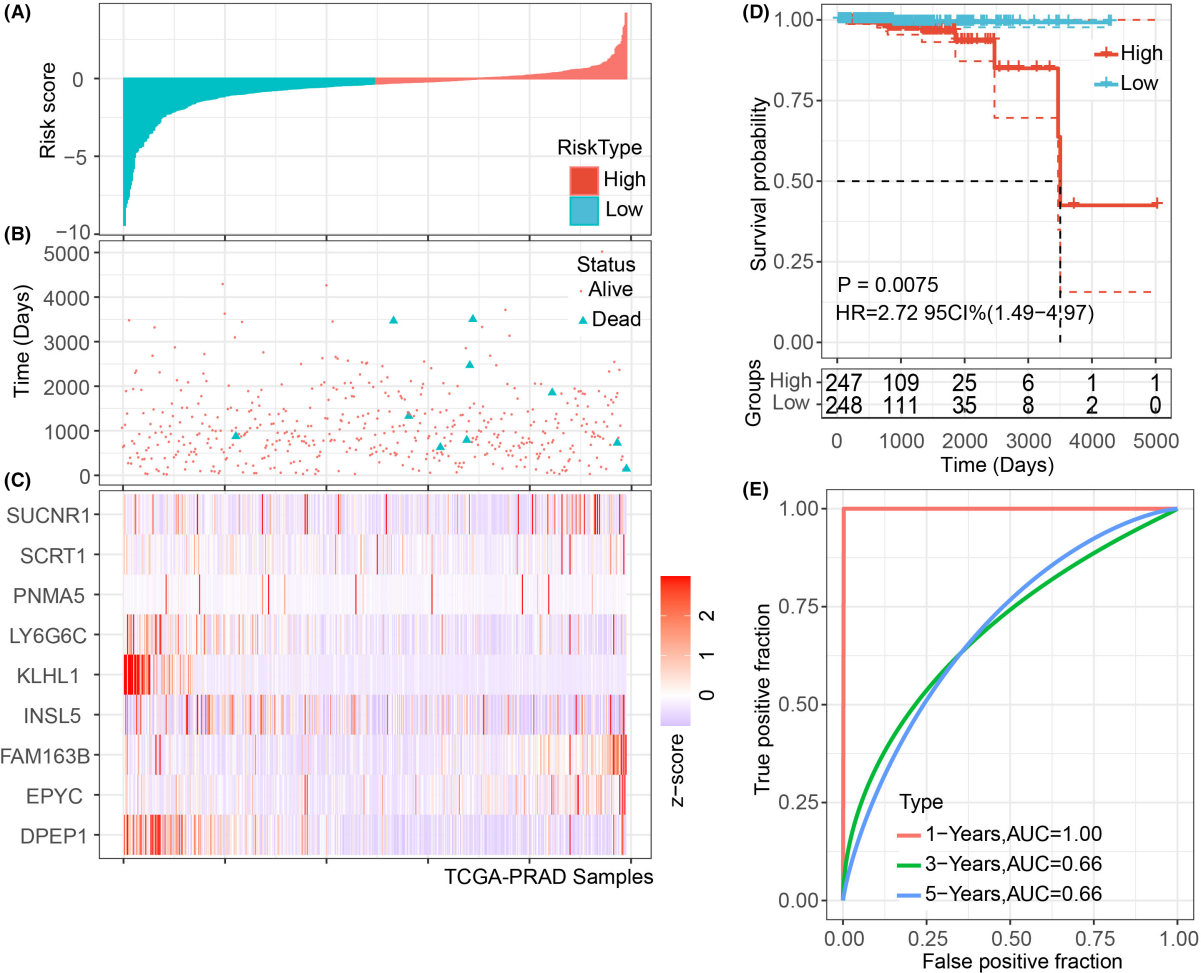
The prediction of OS by the ATRS gene signature of PCa patients in the TCGA‐PRAD cohort. The distribution of risk score and disease status in PCa patients (A, B). The expression levels of the nine age‐ and TME‐related genes (EPYC, FAM163B, INSL5, SUCNR1, SCRT1, DPEP1, KLHL1, LY6G6C, and PNMA5) were visualized in the heatmap (C). The PCa patients with high ATRS had shorter OS than patients with low ATRS (D), and the 1‐, 3‐, and 5‐year ROCs showed the good performance of the ATRS gene signature in predicting the OS of PCa patients (E)

**FIGURE 8:**
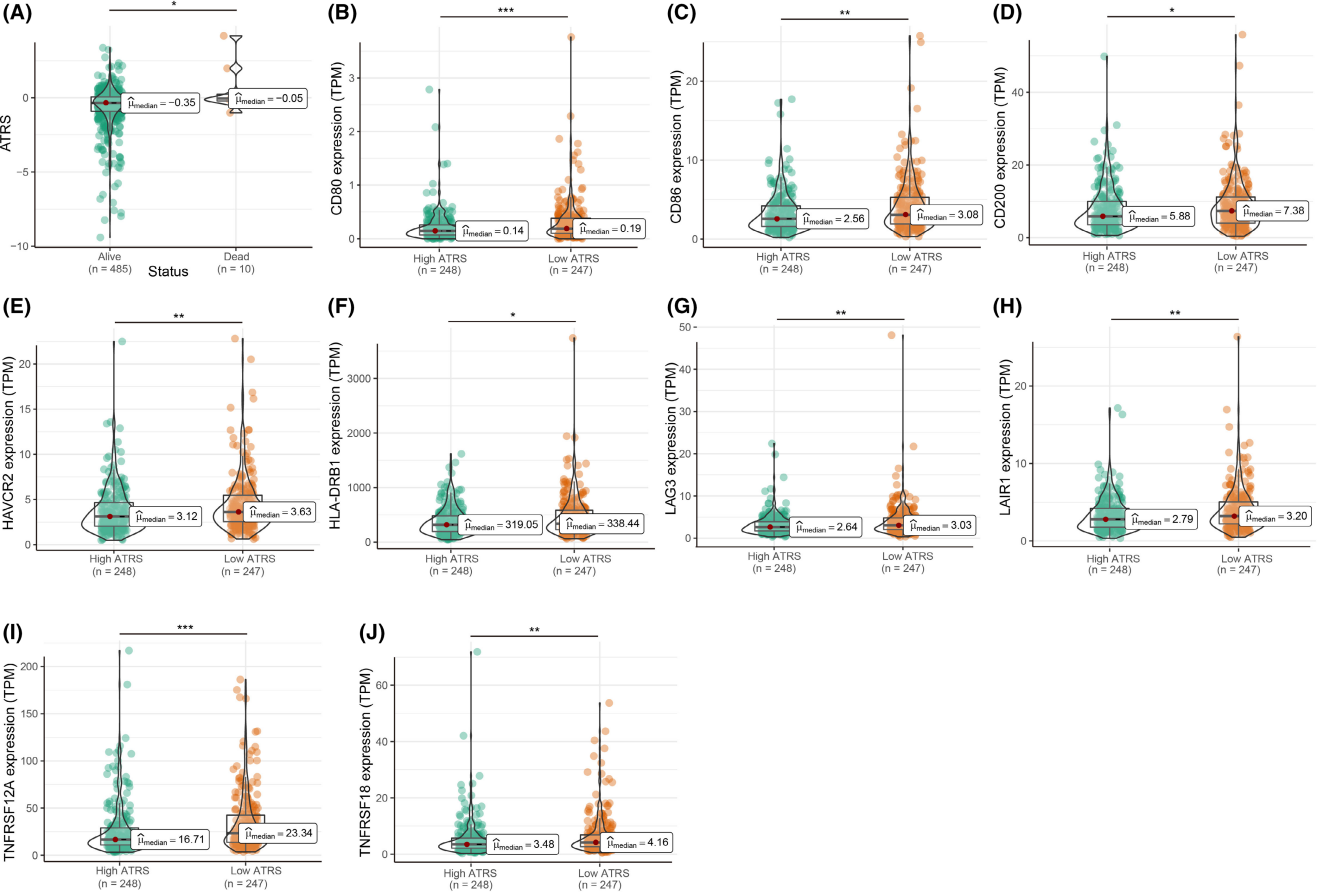
The correlation of ATRS with the expression levels of immune checkpoints. PCa patients with alive status had lower ATRS than dead patients (A), and patients with high ATRS had lower expression levels of CD80, CD86, CD200, HAVCR2, HLA‐DRB1, LAG3, LAIR1, TNFRSF12A, and TNFRSF18 (B–J)

Based on the crucial roles of the nine prognosis‐related DEGs in PCa, we calculated the age‐ and TME‐related risk score (ATRS) of each TCGA‐PRAD samples by Cox regression analysis, and the ATRS was expressed as follows: ATRS = (EPYC expression * 0.770043013606353) + (FAM163B expression * 2.83200842600699) + (INSL5 expression * −0.201150270317484) + (SUCNR1 expression * 0.197919534424295) + (SCRT1 expression * 0.136292272605538) + (DPEP1 expression * ‐1.0685300492505) + (KLHL1 expression * −1.87610207246247) + (LY6G6C expression * −0.880214755865202) + (PNMA5 expression * −0.112025054006857). The risk score and disease status of each TCGA‐PRAD sample were displayed in Figure 7A,B, and the median value was regarded as the cut‐off value. The expression of the nine genes was shown in the heatmap (Figure 7C). The PCa patients with higher risk scores had poorer OS than patients with lower risk scores (Figure 7D). The receiver operating characteristic (ROC) indicated the good performance of the age‐ and TME‐related gene signature in predicting 1/3/5‐year of OS, with an area under the curve (AUC) of 1.00, 0.66, 0.66 (Figure 7E). Additionally, PCa patients with dead status had higher risk scores than patients with alive status (Figure 8A).

### 3.6 ATRSand immune‐related characteristics of PCapatients

The associations between ATRS and 34 types of immune checkpoints were analyzed, and we found that PCa patients with higher ATRS had lower levels of CD80, CD86, CD200, HAVCR2, HLA‐DRB1, LAG3, LAIR1, TNFRSF12A, and TNFRSF18 (Figure 8B–J). Only 44.4% of PCa patients with high ATRS responded to ICB therapy, while 53.0% of patients with low ATRS responded to ICB therapy based on the ImmuoCellAl database (Figure S10A). Moreover, PCa patients with higher ATRS had lower CYT scores, while no significant difference in neoantigens and tumor mutation burdens was observed between high and low ATRS patients (Figure S10B–E), and the correlation of neoantigens and tumor mutations of PRAD was displayed in Figure S10F. For IPS, the correlation of IPS, IPS PD1 blocker, IPS CTLA4, and IPS PD1 and CTLA4 with ATRS was not observed in PCa patients (Figure S10G–J).

### 3.7 Establishment of ATRS‐based OS‐predicting nomogram for PCapatients

**FIGURE 9:**
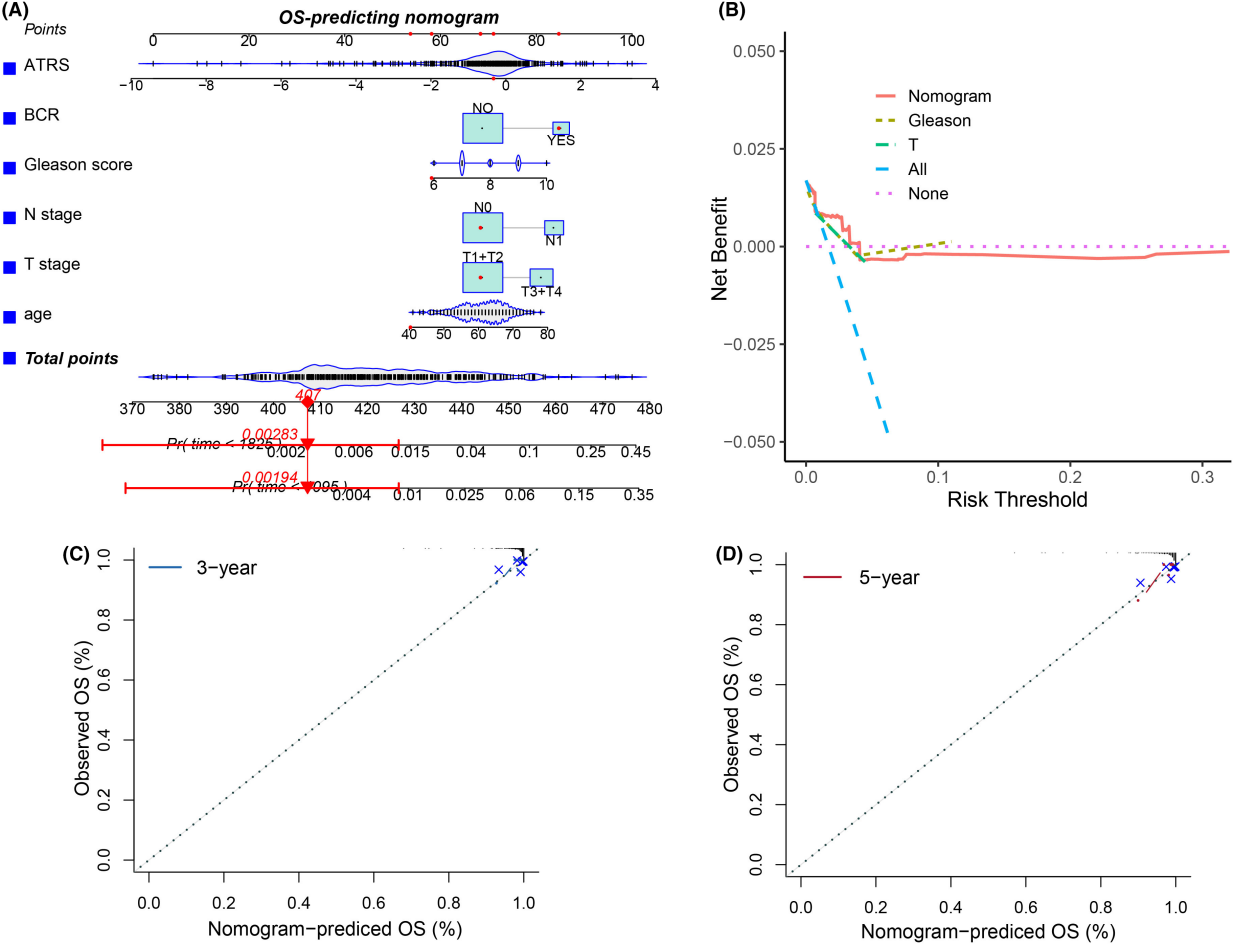
Establishment of the ATRS‐based OS‐predicting nomogram. The ATRS‐based OS‐predicting nomogram was built by integrating ATRS, BCR, Gleason score, N stage, T stage, and age (A), and the decision curve analysis and calibration curve showed the good performance of the ATRS‐based nomogram (B–D)

Based on the significant roles of the ATRS gene signature in PCa, the ATRS‐based nomogram was established to predict the OS of PCa patients. Because age, Gleason score, biochemical recurrence (BCR), T and N stage play important roles in the progression of PCa, hence, these five variables were also integrated into the ATRS‐based nomogram (Figure 9A), which showed good performance in OS prediction, with a concordance index (C‐index) of 0.85 (0.75, 0.95). The decision curve analysis found that the established nomogram performed better than the Gleason score and T stage alone, respectively, and the calibration curve also indicated the performance of the ATRS‐based nomogram (Figure 9B–D).

## 4 DISCUSSION

The current study was devoted to exploring the associations among age, TME, and PCa, and the ATRS gene signature was established based on the nine age‐ and TME‐related DEGs. The main findings were as follows: (1) the OS of PCa patients declined with age, and age was correlated with the clinical characteristics of PCa patients, including tumor stage, T stage, Gleason score, etc.; (2) age was positively correlated with stromal, immune, and ESTIMATE scores, and PCa patients with higher stromal, immune, and ESTIMATE score had higher Gleason score and advanced pathological characteristics; (3) compositions of immune cells altered with age in PCa patients; (4) nine age‐ and TME‐related DEGs were identified, and the ATRS of each TCGA‐PRAD patient was calculated based on these nine genes, which was regarded as the age‐ and TME‐related gene signature; (5) the ATRS was associated with the expression of immune checkpoints and intratumoral cytolytic activity. Taken together, our findings indicated age and TME may play important roles in the development of PCa, which deserved further investigation.

With the increased incidence of PCa and the easy progression to castrate‐resistant prostate cancer (CRPC) after androgen deprivation therapy (ADT), PCa puts a heavy financial burden on males, especially for the elderly.^29^ For advanced PCa, ADT remains the basic treatment, and new strategic managements for CRPC have been developed, including enzalutamide, abiraterone, radium‐223, sipuleucel‐T, etc.^30^ Although more effective treatment has been applied to clinical practice, patients ≥75 years diagnosed as de novo mPCa had shorter PCa–specific survival than patients ≤54 years.^3^ Moreover, PCa patients ≥75 years tended to experience advanced disease compared to younger patients, which led to the poorer outcome and an increased risk of PCa‐related death.^31^, ^32^ In this study, we found that age was correlated with the OS, PSA level, tumor stage, T stage, N stage, Gleason score, and nerve invasion of PCa patients in three cohorts, and the OS of PCa patients was decreased while PSA level was increased with age in the SEER cohort. Consistently, Humphreys et al reported that age was associated with PCa patients' OS and the time from diagnosis to progression to CRPC,^32^ which was a prognostic factor for OS and DSS after receiving ADT.^6^ Therefore, age was associated with the treatment of PCa.

Recently, TME is proposed as a new therapeutic target for tumors.^9^ The immune cells in the TME play vital roles in the tumor progression, for example, tumor‐associated macrophages (TAMs) and myeloid‐derived suppressor cells (MDSCs) promote tumor invasion and contribute to therapeutic inefficacy in many cancer types, and cancer‐associated fibroblasts (CAFs) enhance tumorigenesis by secreting growth factors and activating NF‐κB signaling pathway.^8^ For PCa, tumor‐infiltrating immune cells exerted important roles in its progression, metastasis, chemotherapy, and radiotherapy‐related resistance.^33^, ^34^ The infiltration and activities of immune cells in the tumor immune microenvironment of PCa were altered after ADT.^35^ In the immunosenescent processes, the cytotoxic abilities of NK cells, dendritic cells (DCs), and effector T cells were reduced, and the antigen capture and presentation abilities of DCs were attenuated, causing a reduction in immune surveillance.^13^, ^14^ Moreover, the structure of the aged TME changed, which promoted the development of tumors.^15^ Based on the significant effects of age and TME on PCa, we investigated the association between age, TME, and PCa. We found that age was positively associated with stromal, immune, and ESTIMATE score, and PCa patients with higher stromal, immune, and ESTIMATE scores had advanced pathological characteristics, including Gleason score, N stage, and residual tumor. Additionally, our results showed that infiltration of immune cells in the TME of PCa altered with age, which demonstrated the significant role of age and TME in the progression of PCa, and the underlying molecular mechanisms deserved in‐depth study.

Other previous studies have developed several Gleason score‐related gene signatures to accurately predict the lethality of PCa with Gleason score 7 and to maximize the management of PCa,^36^, ^37^ and 157 and 30 genes were integrated into these two signatures, respectively. Additionally, a 35 gene signature was built to achieve better treatment decisions for PCa.^38^ These three gene signatures mentioned above integrated more than 30 genes, making it difficult to apply in clinics with a high cost to patients. Shao et al establish a prognostic gene signature based on six genes to predict the infiltration of immune cells, OS, and biochemical recurrence of PCa, while age was not taken into account.^39^ Based on the crucial roles of age and TME in PCa, we identified nine age‐ and TME‐related DEGs, and the ATRS of each TCGA‐PRAD patient was calculated based on the identified nine genes. The ATRS gene signature was negatively associated with OS of PCa patients, which showed good performance in predicting the PCa patients' OS. Therefore, we built an age‐ and TME‐related gene signature to predict the OS of PCa patients.

PCa was defined as a “cold” tumor with a low level of immune infiltration and minimal T‐cell infiltration, indicating that PCa patients' response to mono‐immunotherapy was poor, and immunotherapy combined with other therapies has been performed in some clinical trials.^40^ We explored the potential relationship between ATRS and immunotherapy response, and PCa patients with higher ATRS had lower expression levels of immune checkpoints. Similarly, only 44.4% PCa patients with high ATRS responded to ICB therapy, while 53.0% of patients with low ATRS responded to ICB therapy. Hence, the ATRS gene signature may be associated with the response to ICB therapy. Tumor mutational burden (TMB) has regulatory roles in immune responses by the production of neoantigens,^41^ which may affect patients' response to ICB. In this study, we found the association between TBM and the production of neoantigens in PCa, and PCa patients with high ATRS have lower immune cytolytic activities of the intratumoral immune infiltration than patients with low ATRS. Hence, our findings indicated age and TME may play important roles in the development of PCa, and the established ATRS gene signature was associated with OS and immune characteristics of PCa patients, which deserved further investigations. To better apply the ATRS gene signature into clinical practice, we established the ATRS‐based nomogram to predict the outcomes of PCa patients, which performed well in OS‐predicting. Altogether, the ATRS gene signature played important role in the treatment and outcome prediction of PCa.

The main shortcomings of the current study were that the effects of the nine identified age‐ and TME‐related DEGs on the progression of PCa were not assessed by functional experiments, and further study was needed to explore their roles in PCa. The established ATRS gene signature and ATRS‐based nomogram were not externally validated by the real‐world cohort because the corresponding RNA‐seq data and survival data were not obtained, which restricted the widespread use of the ATRS gene signature. In the next step, we would collect the PCa tissues and perform RNA‐sequence to validate the established ATRS gene signature and ATRS‐based nomogram. Additionally, the association between ATRS and the response to ICB was predicted by the online tool, which deserved further study to examine the predictive value of ATRS on ICB response.

## 5 CONCLUSIONS

In summary, we found that age and TME were associated with the clinical characteristics of PCa patients, and the ATRS gene signature based on the nine identified age‐ and TME‐related DEGs was established, which was negatively correlated with OS of PCa patients.

## CONFLICT OF INTEREST

The authors declare that there is no conflict of interest regarding the publication of this paper.

## AUTHORS’ CONTRIBUTION

LC and MZ analyzed data, drew illustrations, and wrote the manuscript; JZ, LZ, and CL designed the study and revised the manuscript. All authors contributed to this manuscript. All authors read and approved the final manuscript.

## ETHICAL APPROVAL STATEMENT

This study was approved by the committee of The First Affiliated Hospital of Anhui Medical University (PJ 2021‐14‐23).

## Supporting information


Figure S1

Figure S2

Figure S3

Figure S4

Figure S5

Figure S6

Figure S7

Figure S8

Figure S9

Figure S10

Table S1

Table S2

## Data Availability

The data in the current study are available from the corresponding author on reasonable request.
