# Supplementary figures and images for: Establishment of an age‐ and tumor microenvironment‐related gene signature for survival prediction in prostate cancer
Source: Cancer Med. 2022 May 9;11(22):4374–88. doi: 10.1002/cam4.4776 (PMC9678094; doi:10.1002/cam4.4776)

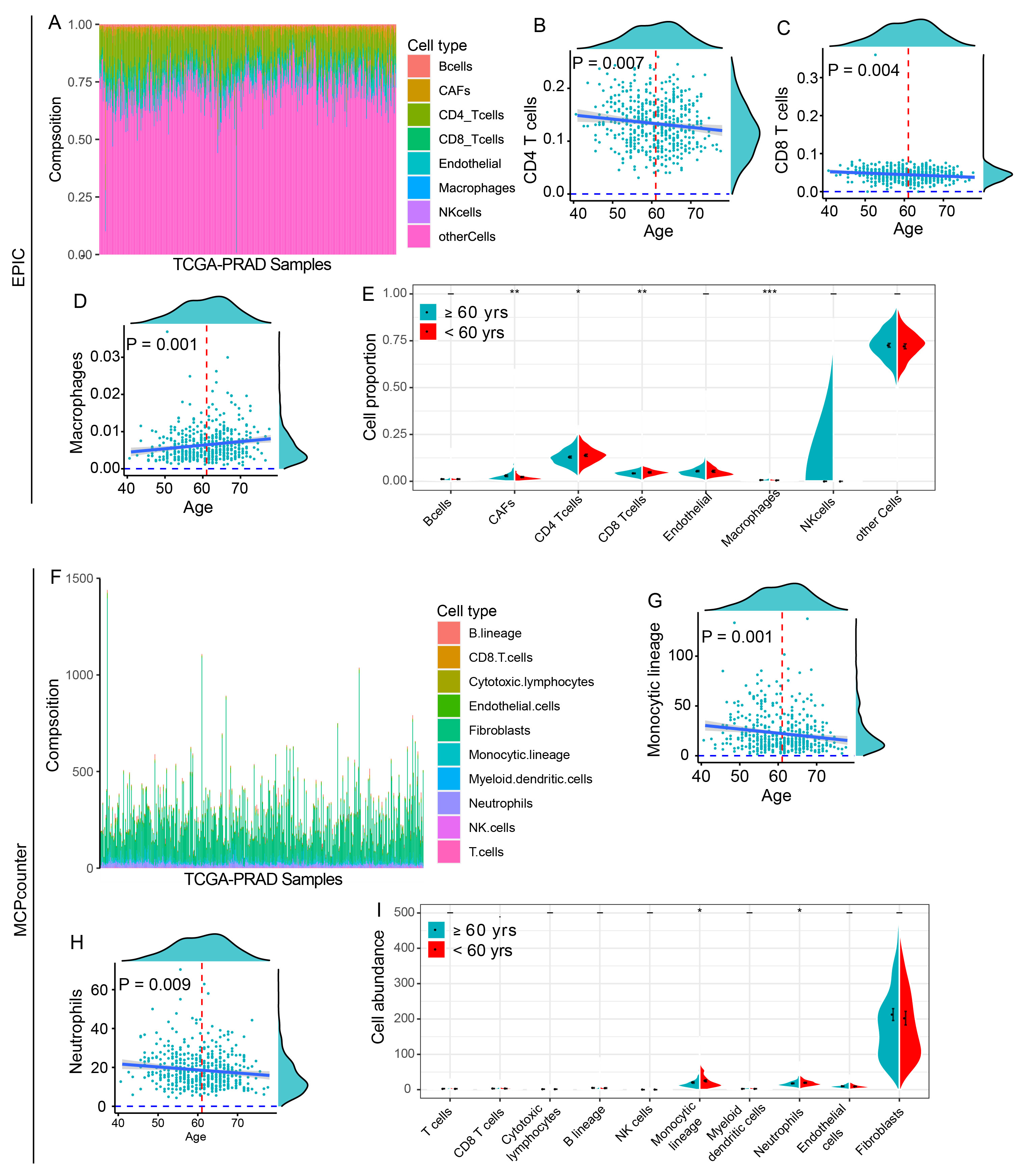

Supplement: Supplementary file 1 — Figure S1 [file CAM4-11-4374-s002.jpg]

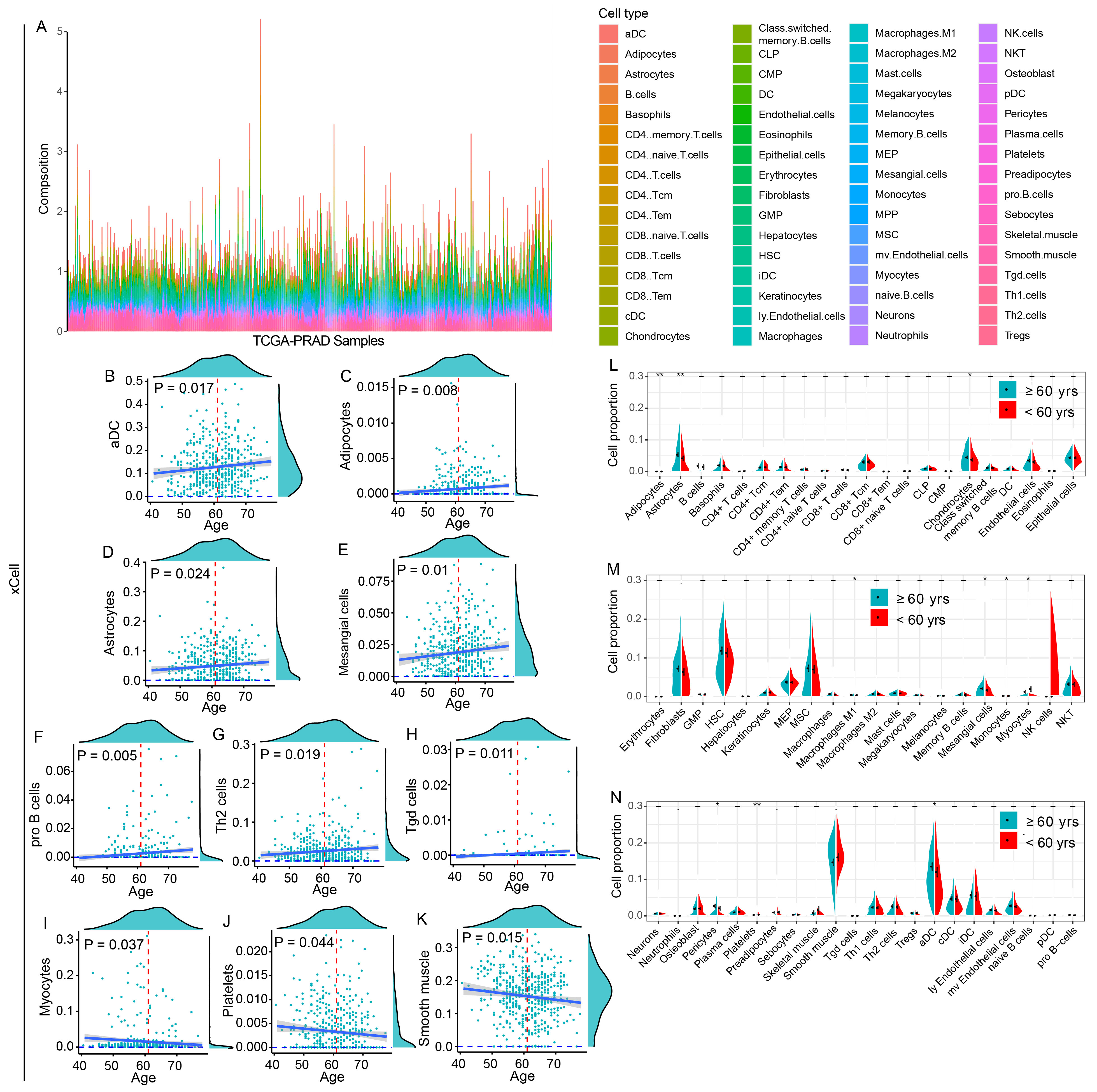

Supplement: Supplementary file 2 — Figure S2 [file CAM4-11-4374-s005.jpg]

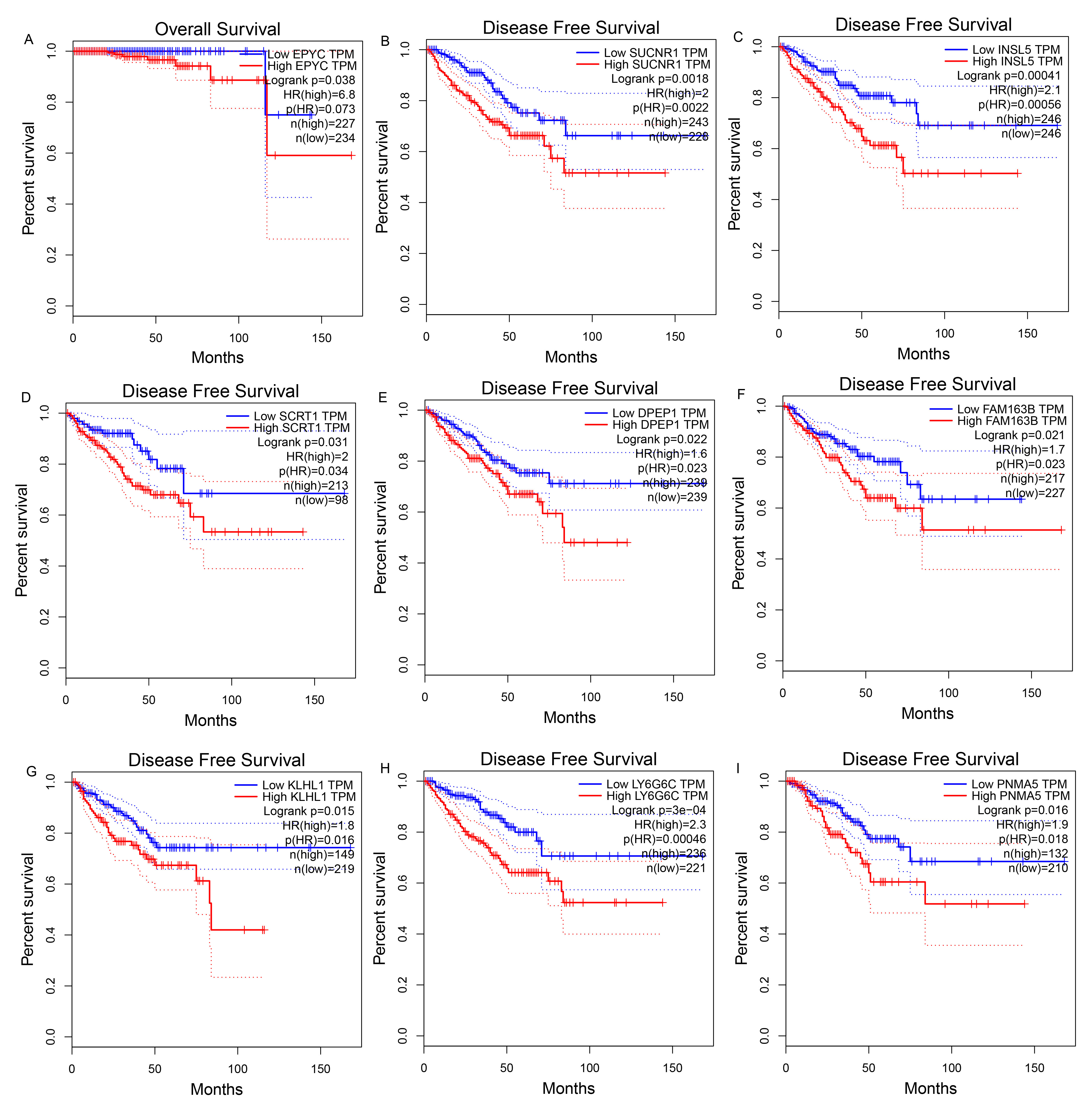

Supplement: Supplementary file 3 — Figure S3 [file CAM4-11-4374-s009.jpg]

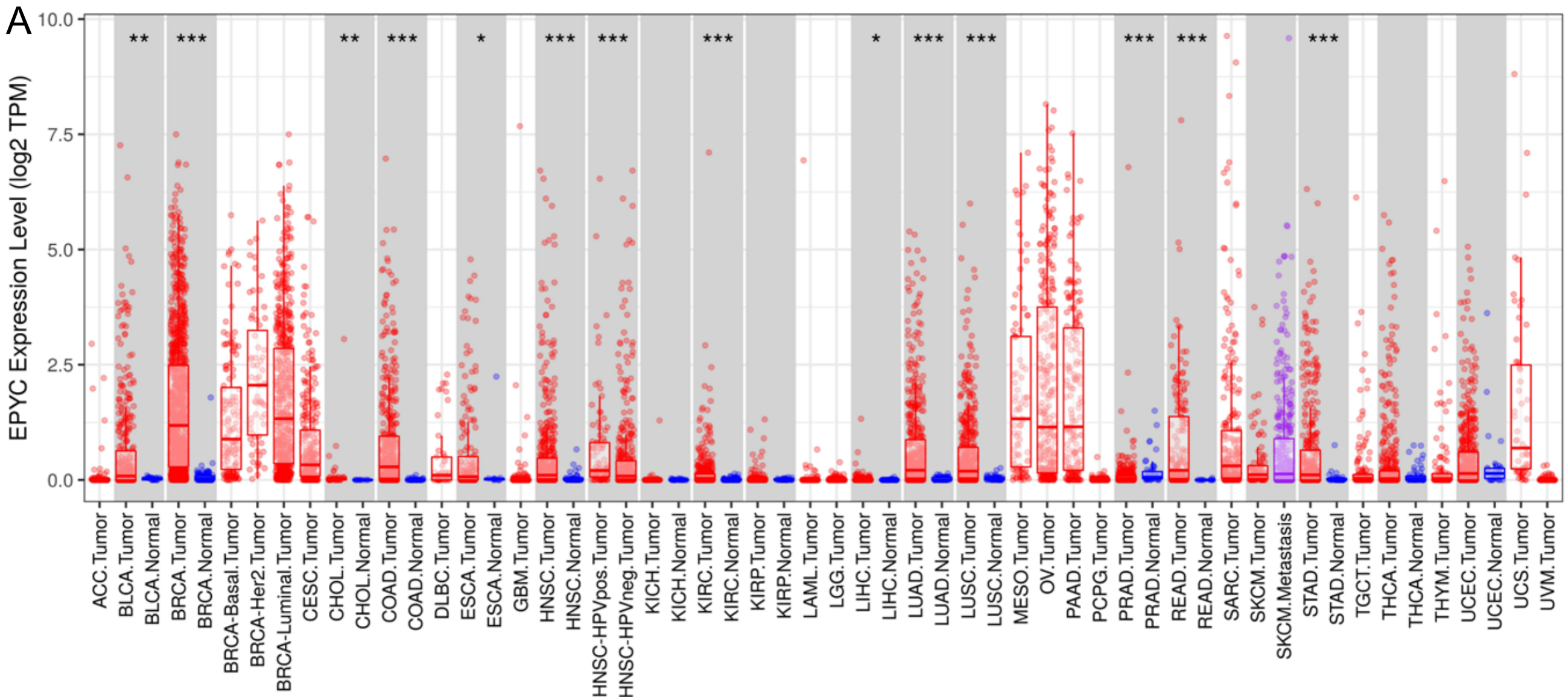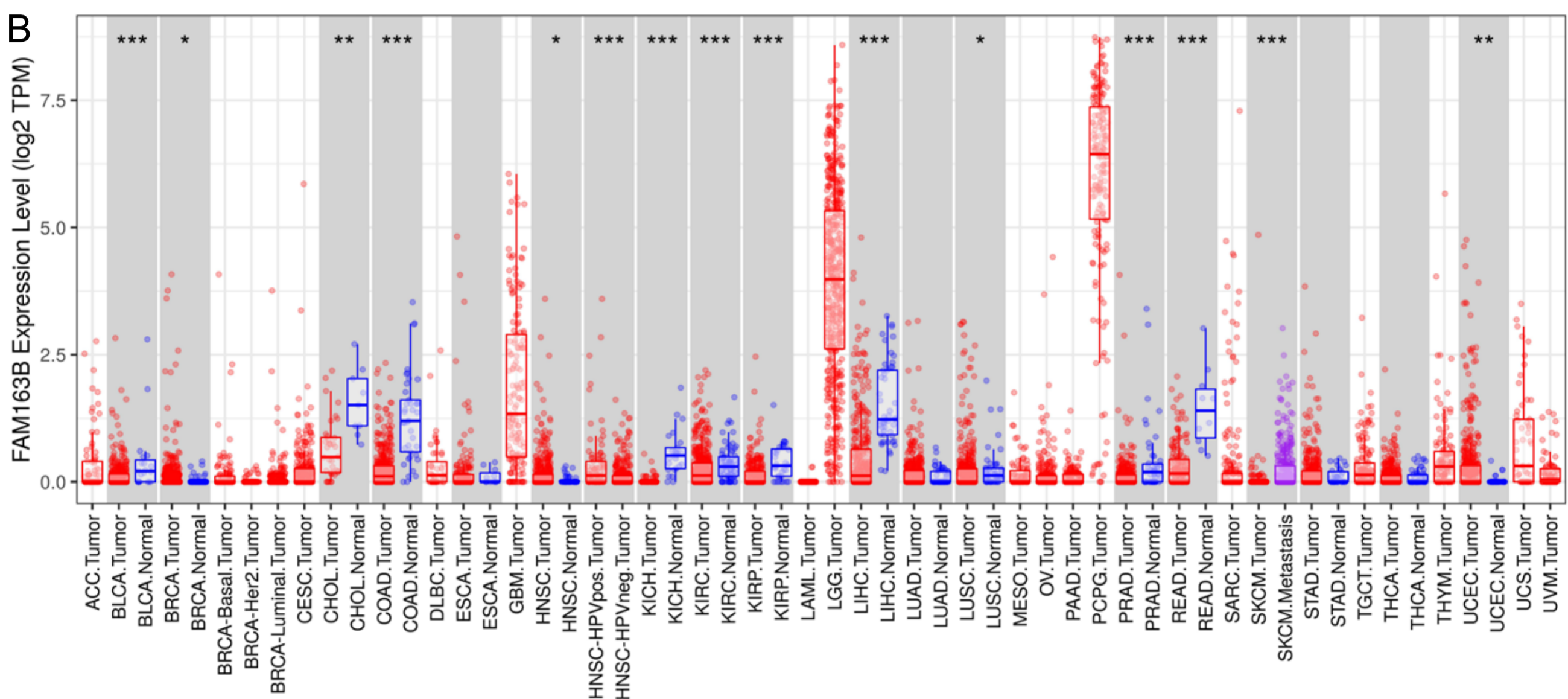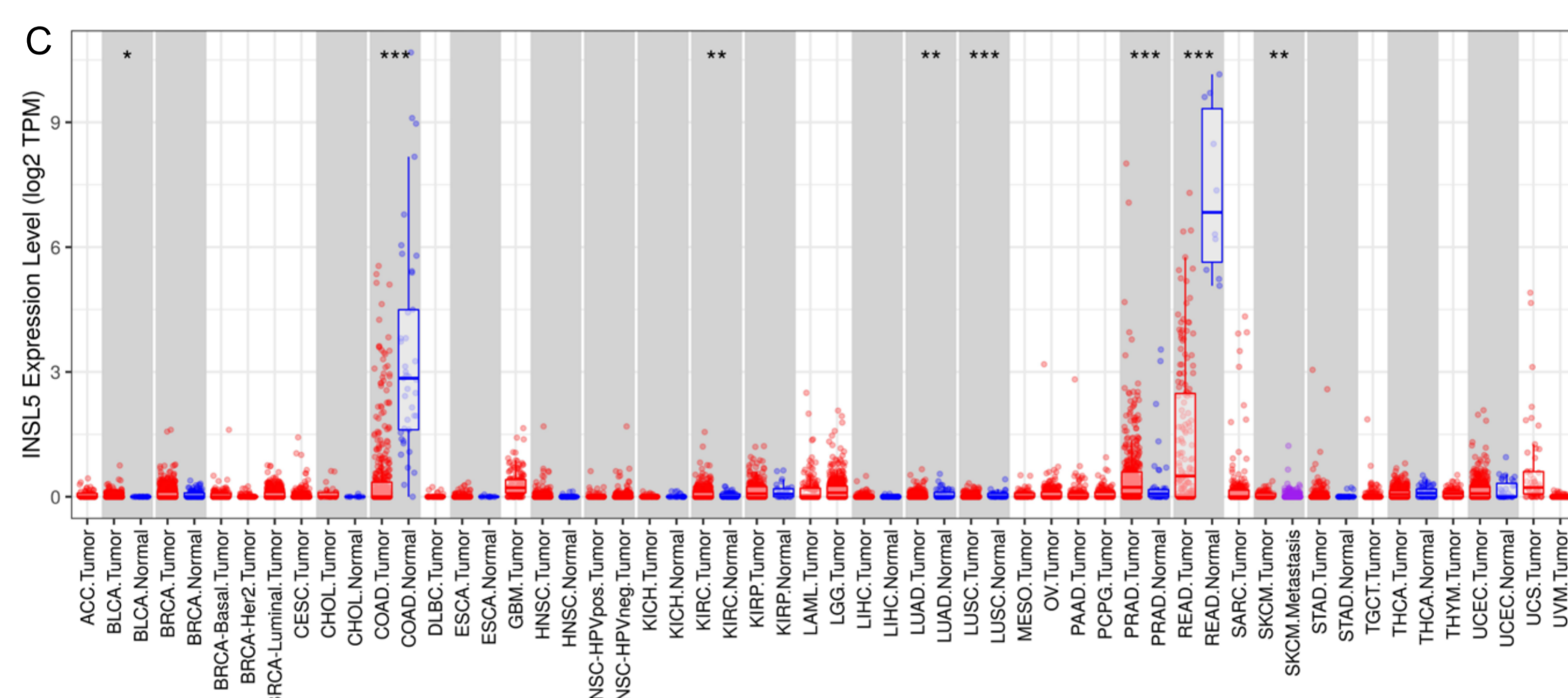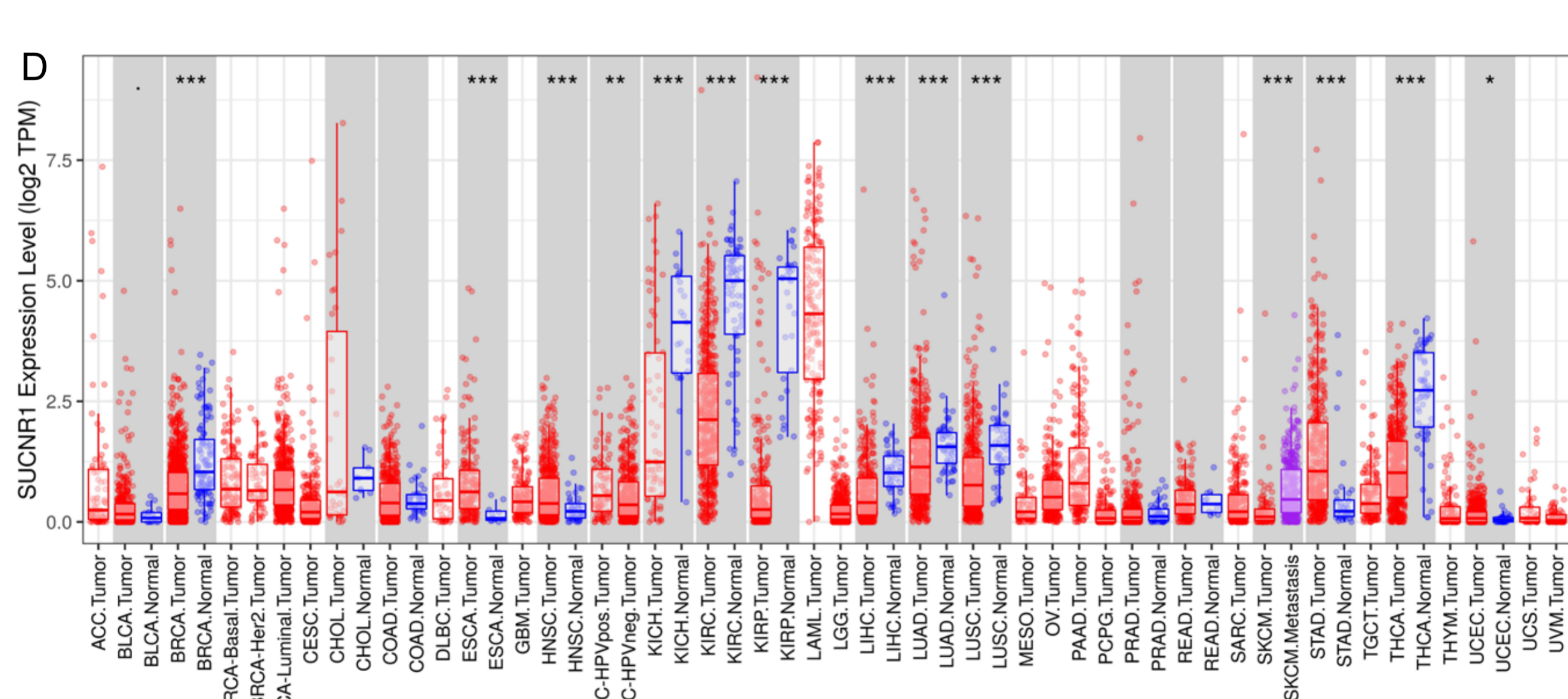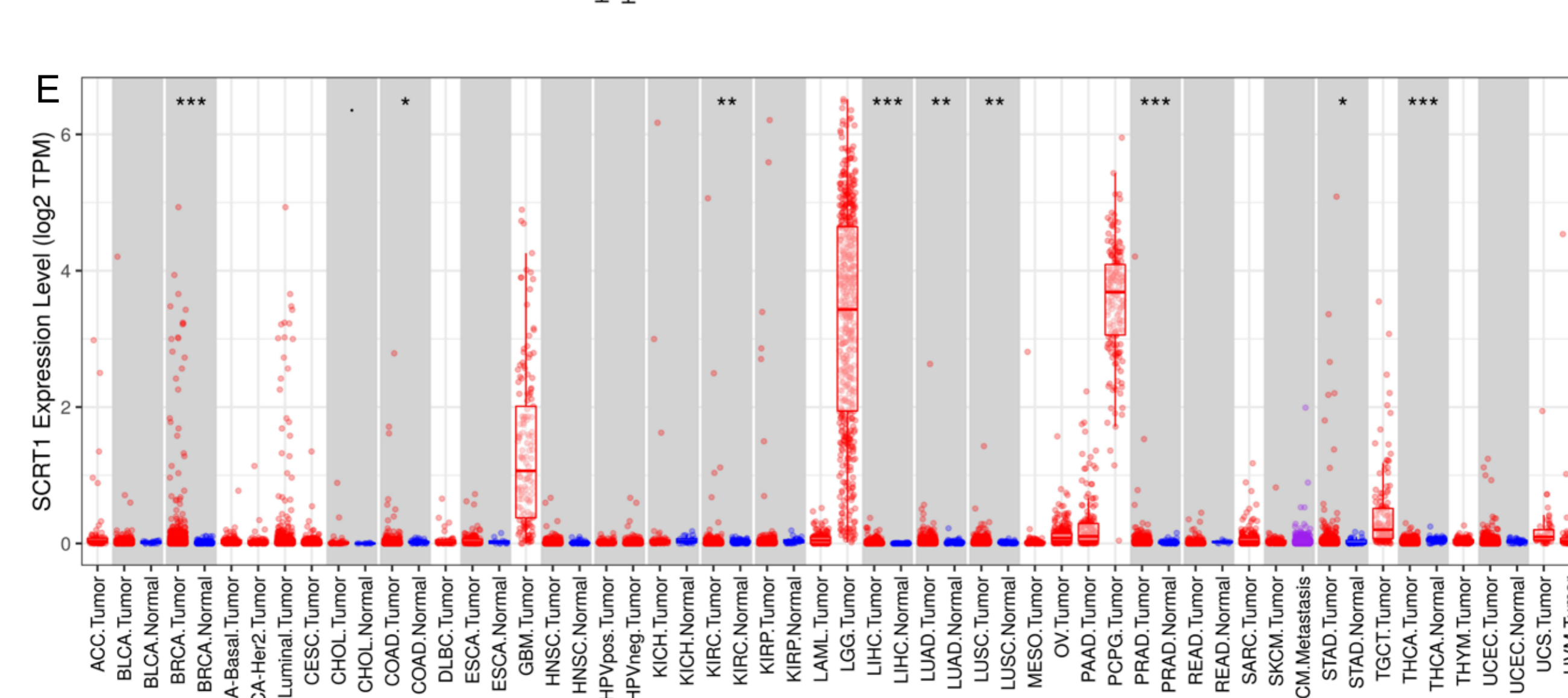

Supplement: Supplementary file 4 — Figure S4 [file CAM4-11-4374-s001.pdf]

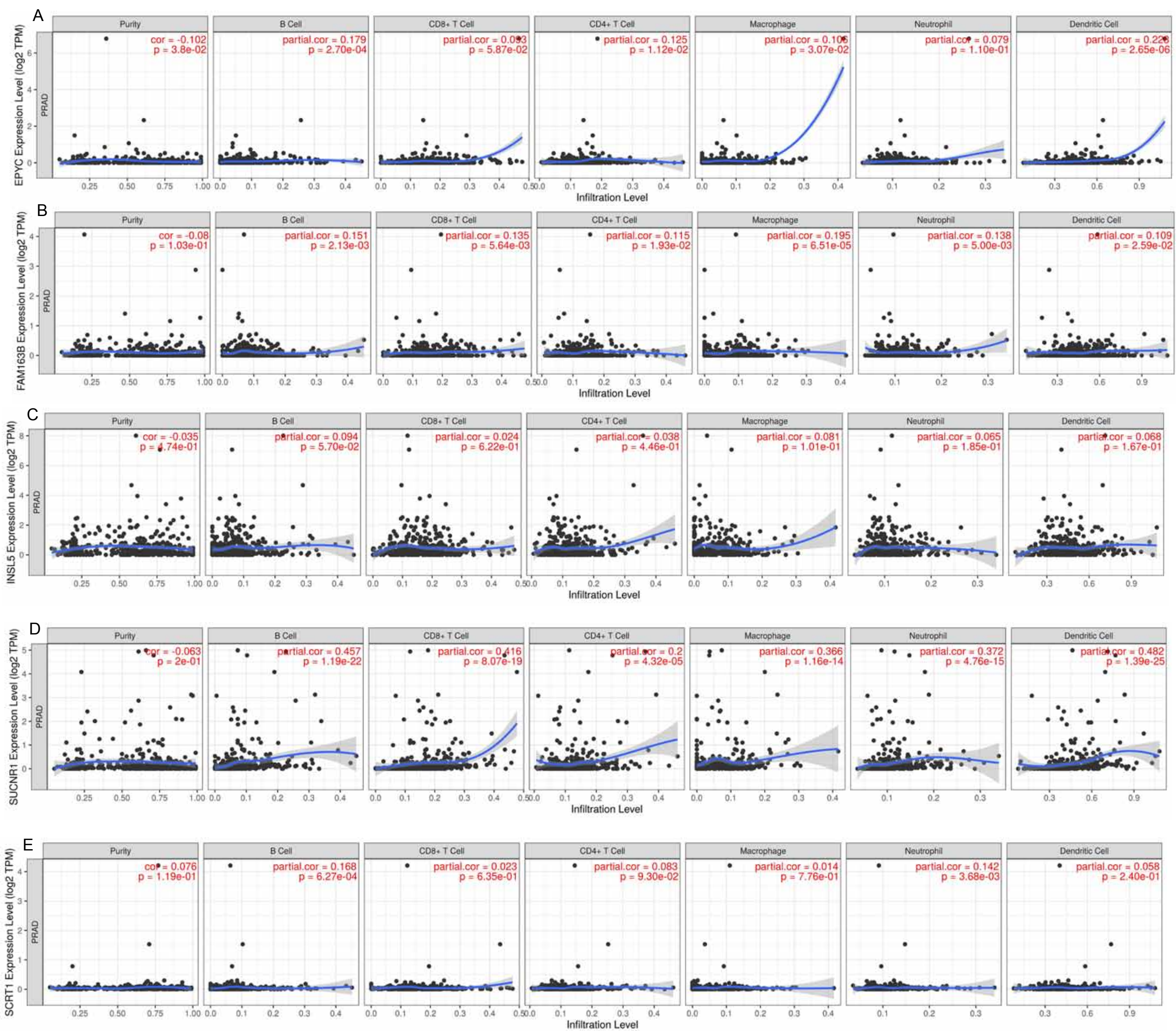

Supplement: Supplementary file 6 — Figure S6 [file CAM4-11-4374-s008.pdf]

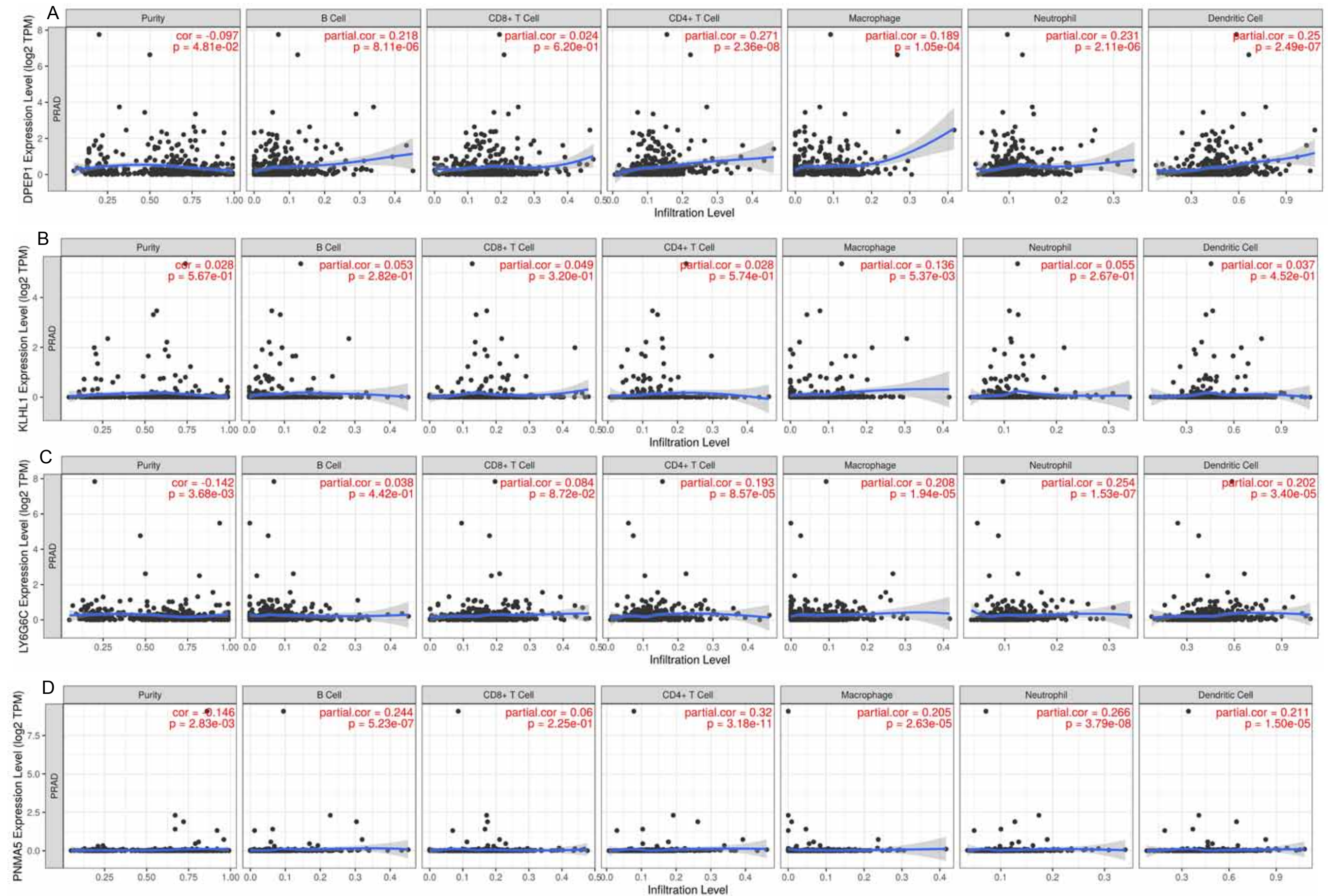

Supplement: Supplementary file 7 — Figure S7 [file CAM4-11-4374-s004.pdf]

# EPYC

A

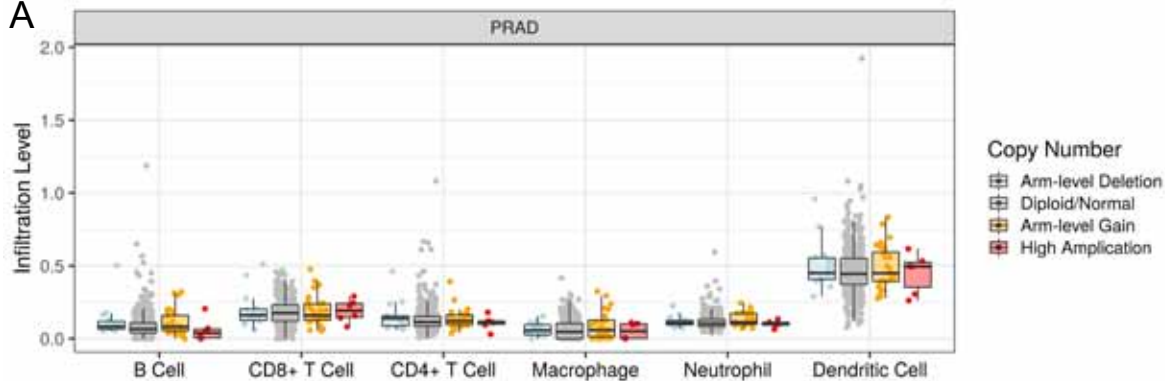

# FAM163B

B

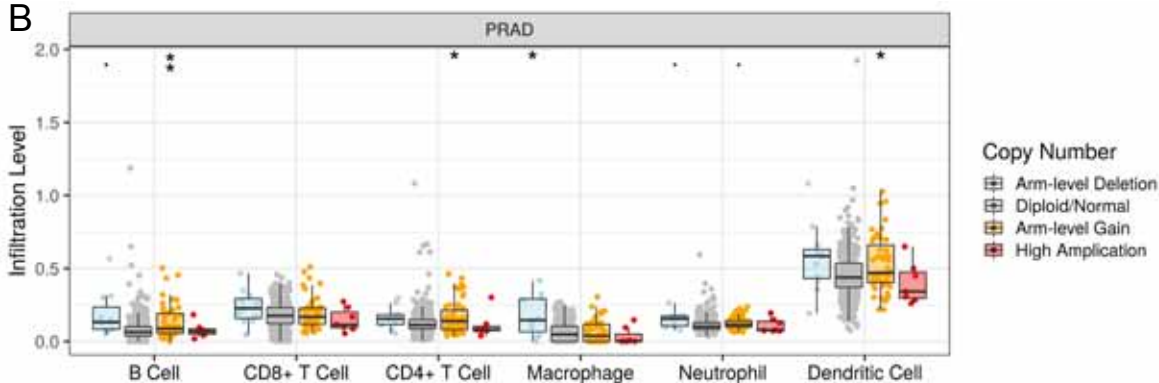

# INSL5

C

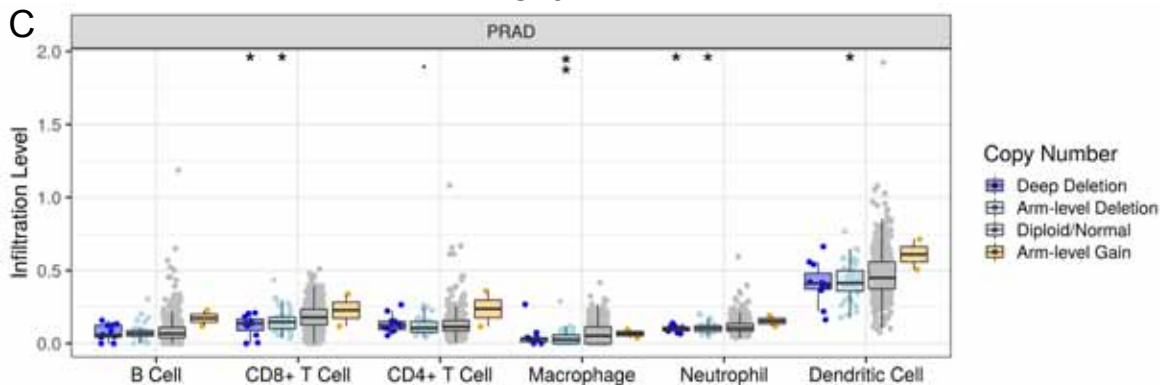

# SUCNR1

D

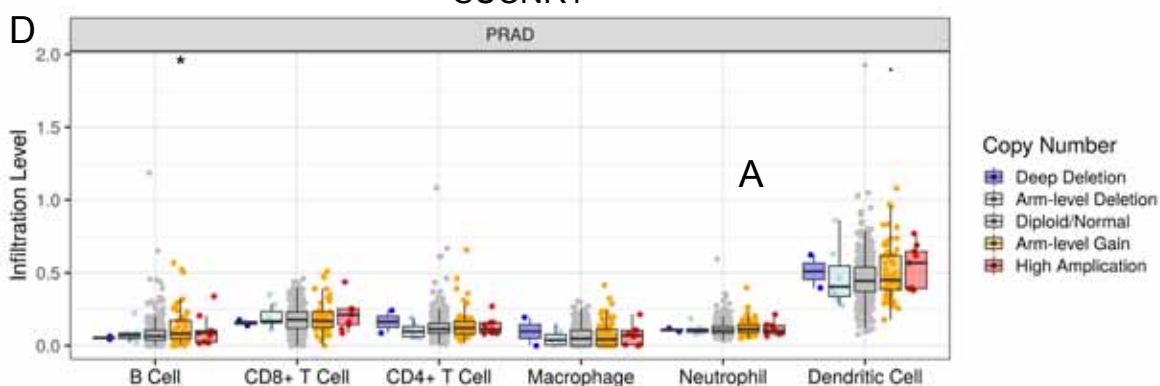

# SCRT1

E

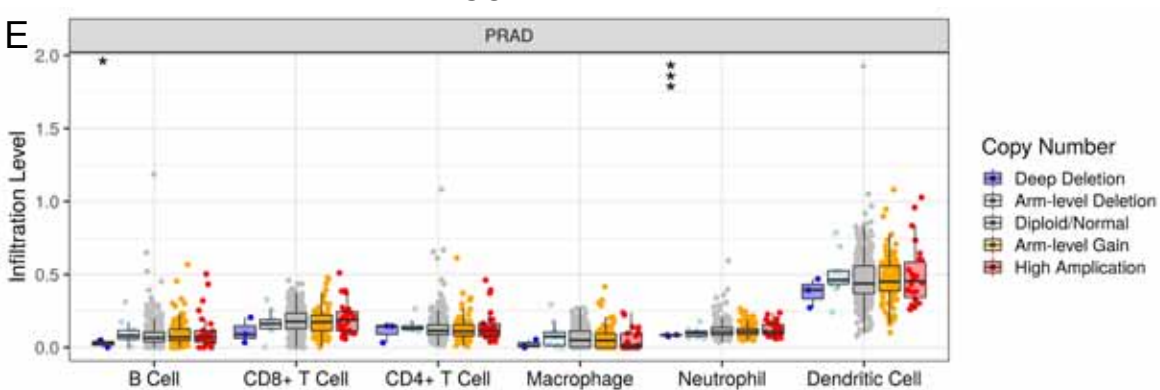

Supplement: Supplementary file 8 — Figure S8 [file CAM4-11-4374-s007.pdf]

# DPEP1

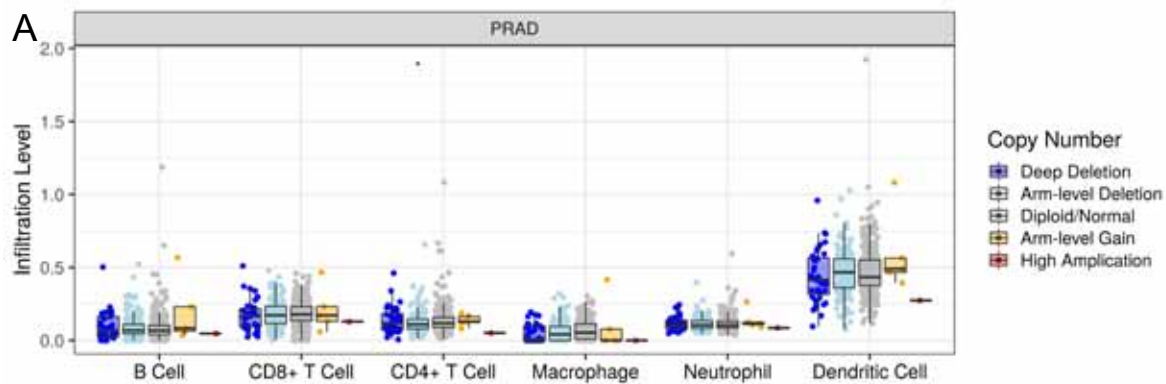

# KLHL1

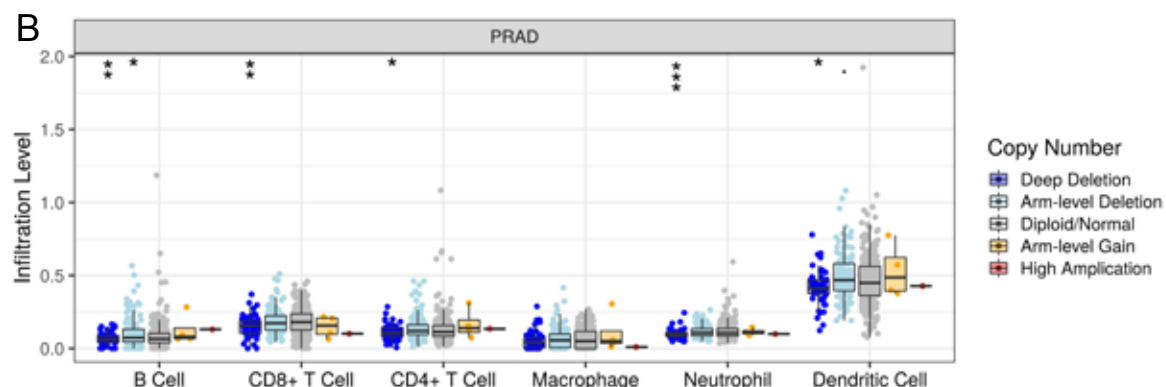

# LY6G6C

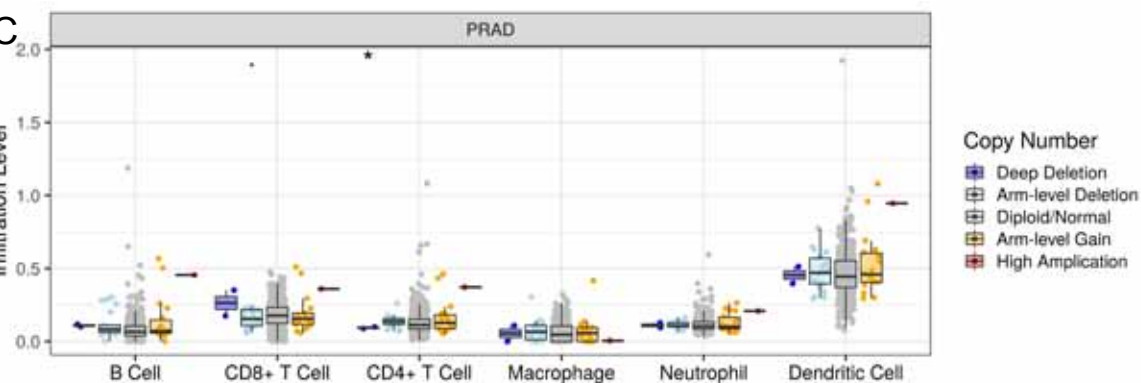

# PNMA5

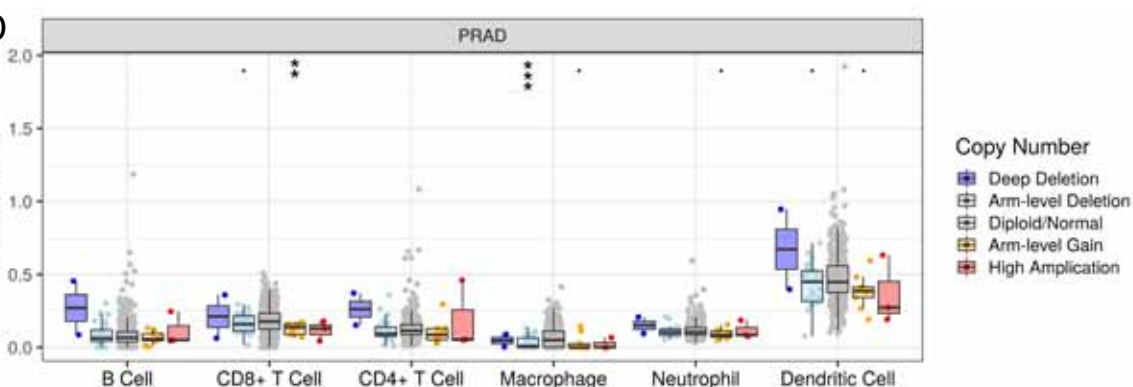

Supplement: Supplementary file 9 — Figure S9 [file CAM4-11-4374-s011.pdf]

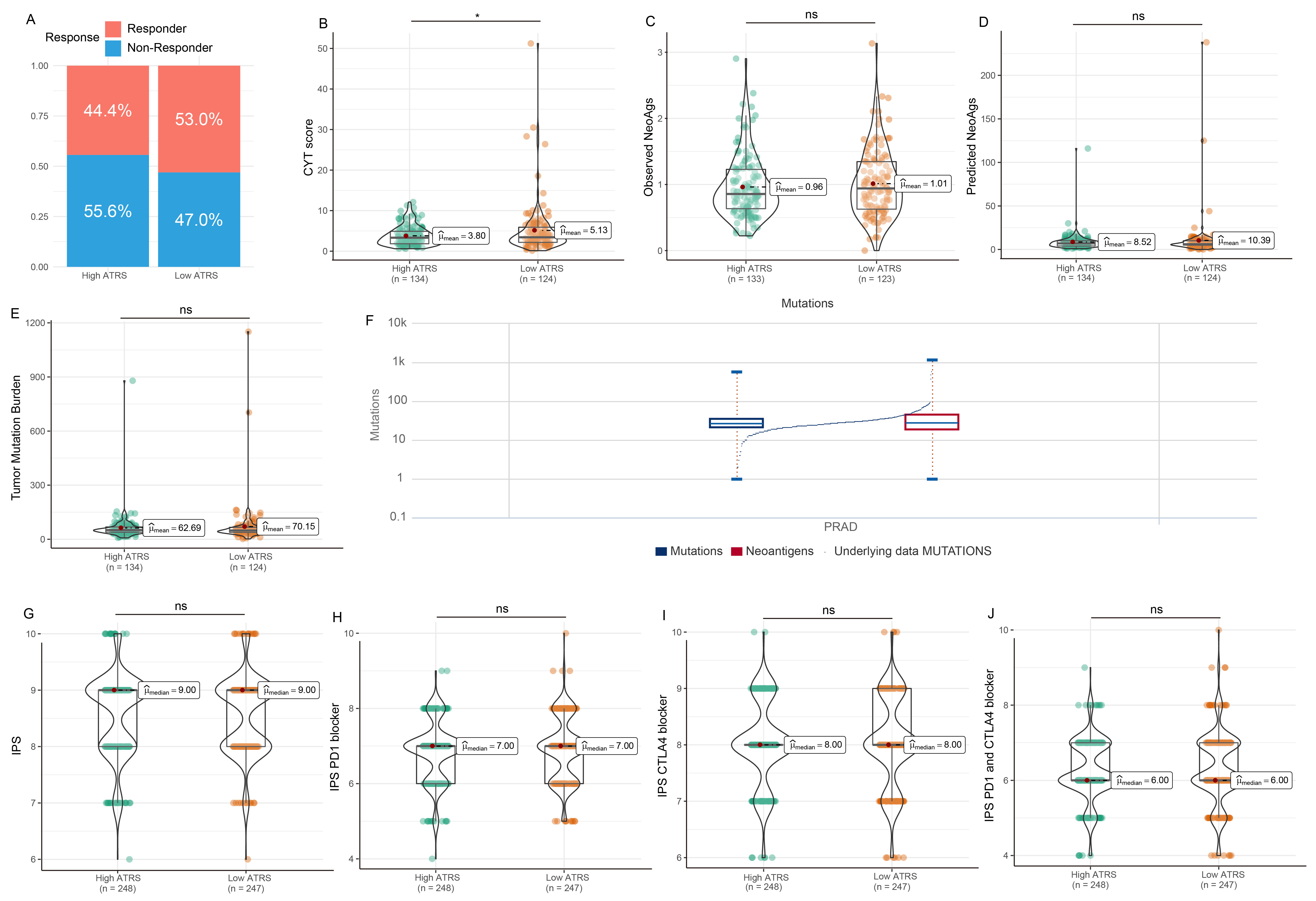

Supplement: Supplementary file 10 — Figure S10 [file CAM4-11-4374-s012.jpg]
