# Supplementary material for: Establishment of an age‐ and tumor microenvironment‐related gene signature for survival prediction in prostate cancer
Source: Cancer Med. 2022 May 9;11(22):4374–88. doi: 10.1002/cam4.4776 (PMC9678094; doi:10.1002/cam4.4776)
Supplement: Supplementary file 11 — Table S1 [file CAM4-11-4374-s003.docx]

**Supplemental Table 1. Characteristics of prostate cancer patients at different age stages in SEER-PRAD cohort.**

|  | Age (years) |  |  |  |  |
| --- | --- | --- | --- | --- | --- |
| Characteristic | < 60 | ≥ 60 | Total | χ^2^ | *P* |
|  | n = 11643 | n = 18623 | 30266 |  |  |
| Race, no. (%) |  |  |  | 436.452 | **< 0.001** |
| White | 9069 (77.9) | 15427 (82.8) | 24496 |  |  |
| Black | 1948 (16.7) | 1783 (9.6) | 3731 |  |  |
| Other | 481 (4.1) | 1283 (6.9) | 1764 |  |  |
| Unknown | 145 (1.2) | 130 (0.7) | 275 |  |  |
| AJCC stage, no. (%) |  |  |  | 330.919 | **< 0.001** |
| I | 725 (6.2) | 733 (3.9) | 1458 |  |  |
| II | 8085 (69.4) | 11676 (62.7) | 19761 |  |  |
| III | 2366 (20.3) | 5332 (28.6) | 7698 |  |  |
| IV | 467 (4.0) | 882 (4.7) | 1349 |  |  |
| T stage, no. (%) |  |  |  | 274.826 | **< 0.001** |
| T1 | 11 (0.1) | 31 (0.2) | 42 |  |  |
| T2 | 8855 (76.1) | 12506 (67.2) | 21361 |  |  |
| T3 | 2734 (23.5) | 6009 (32.3) | 8743 |  |  |
| T4 | 43 (0.4) | 77 (0.4) | 120 |  |  |
| N stage, no. (%) |  |  |  | 10.238 | **0.006** |
| N0 | 11221 (96.4) | 17816 (95.7) | 29037 |  |  |
| N1 | 422 (3.6) | 805 (4.3) | 1227 |  |  |
| Nx | 0 (0.0) | 2 (0.0) | 2 |  |  |
| M stage, no. (%) |  |  |  | 1.012 | 0.314 |
| M0 | 11623 (99.8) | 18581 (99.8) | 30204 |  |  |
| M1 | 20 (0.2) | 42 (0.2) | 62 |  |  |
| Clinical Gleason score, no. (%) |  |  |  | **374.707** | **< 0.001** |
| ≤7 | 10394 (89.3) | 15069 (80.9) | 25463 |  |  |
| > 7 | 1249 (10.7) | 3554 (19.1) | 4803 |  |  |
| Pathological Gleason score, no. (%) |  |  |  | **242.908** | **< 0.001** |
| ≤ 7 | 10627 (91.3) | 15865 (85.2) | 26492 |  |  |
| > 7 | 1016 (8.7) | 2758 (14.8) | 3774 |  |  |
| PSA level, ng/mL, no. (%) |  |  |  | **72.907** | **< 0.001** |
| < 10 | 9547 (82.0) | 14676 (78.8) | 24223 |  |  |
| 10-20 | 1466 (12.6) | 3010 (16.2) | 4476 |  |  |
| > 20 | 630 (5.4) | 937 (5.0) | 1567 |  |  |

IQR = interquartile range; PSA = prostate-specific antigen; AJCC = American Joint Committee on Cancer; M1a = nonregional lymph nodes; M1b = bone metastasis with or without lymph nodes; M1c = distant metastasis with or without bone and/or lymph node involvement; EBRT = external-beam radiation therapy; NSR = no surgery or radiation therapy; RP = radical prostatectomy; BT = brachytherapy. the Surveillance, Epidemiology and End Results Database
