# Supplementary material for: Establishment of an age‐ and tumor microenvironment‐related gene signature for survival prediction in prostate cancer
Source: Cancer Med. 2022 May 9;11(22):4374–88. doi: 10.1002/cam4.4776 (PMC9678094; doi:10.1002/cam4.4776)
Supplement: Supplementary file 12 — Table S2 [file CAM4-11-4374-s006.docx]

**Supplemental Table 2. Characteristics of prostate cancer patients at different age stages in our cohort.**

|  | Age (years) |  |  |  |  |
| --- | --- | --- | --- | --- | --- |
| Characteristic | < 60 | ≥ 60 | Total | *t* / χ^2^ | *P* |
|  | n = 68 | n = 373 | 441 |  |  |
| **BMI (kg/m^2^)** | 24.10 ± 3.18 | 23.62 ± 3.01 |  | 1.199 | 0.231 |
| **T stage, no. (%)** |  |  |  | <0.001 | 0.989 |
| T12 | 53 (77.9) | 291 (78.0) | 344 |  |  |
| T34 | 15 (22.1) | 82 (22.0) | 97 |  |  |
| **Pathological Gleason score, no. (%)** |  |  |  | 2.429 | 0.119 |
| ≤ 7 | 37 (54.4) | 240 (64.3) | 277 |  |  |
| > 7 | 31 (45.6) | 133 (35.7) | 164 |  |  |
| **Pathological Primary Gleason score, no. (%)** |  |  |  | 1.633 | 0.201 |
| ≤ 3 | 28 (41.2) | 185 (49.6) | 213 |  |  |
| > 3 | 40 (58.8) | 188 (50.4) | 228 |  |  |
| **PSA level, ng/mL, no. (%)** |  |  |  | 3.049 | 0.218 |
| < 10 | 24 (35.3) | 133 (35.7) | 157 |  |  |
| 10-20 | 27 (39.7) | 113 (30.3) | 140 |  |  |
| > 20 | 17 (25.0) | 127 (34.0) | 144 |  |  |
| **Nerve invasion** |  |  |  | **8.297** | **0.004** |
| Yes | 38 (55.9) | 139 (37.3) | 177 |  |  |
| No | 30 (44.1) | 234 (62.7) | 264 |  |  |
| **Seminal vesicle invasion** |  |  |  | 1.264 | 0.261 |
| Yes | 12 (17.6) | 47 (12.6) | 59 |  |  |
| No | 56 (82.4) | 326 (87.4) | 382 |  |  |
